# Crystallin Alpha B Inhibits Cocaine‐Induced Conditioned Place Preference via the Modulation of Dopaminergic Neurotransmission

**DOI:** 10.1111/adb.70028

**Published:** 2025-03-17

**Authors:** Sun Mi Gu, Daejin Park, Sowoon Seo, Sanghyeon Kim, Young Eun Kim, Maree J. Webster, Heejong Eom, Dohyun Lee, Jin Tae Hong, Sang‐Bae Han, Hye Jin Cha, Jaesuk Yun

**Affiliations:** ^1^ College of Pharmacy Chungbuk National University Cheongju Chungcheongbuk Republic of Korea; ^2^ Stanley Brain Research Laboratory Stanley Medical Research Institute Rockville Maryland USA; ^3^ Laboratory Animal Center Osong Medical Innovation Foundation Cheongju Chungcheongbuk Republic of Korea; ^4^ College of Veterinary Medicine Gyeongsang National University Jinju Gyeongsangnam Republic of Korea

**Keywords:** cocaine relapse, crystallin alpha B, drug addiction, excitatory amino acid transporter 2, nucleus accumbens, oligodendrocytes

## Abstract

Nonneuronal cells mediate neurotransmission and drug addiction. However, the role of oligodendrocytes in stress‐induced cocaine relapses remains unclear. In the present study, we investigated the role of the oligodendrocyte‐abundant molecule crystallin alpha B (CRYAB) in cocaine‐induced conditioned place preference (CPP) relapsed by restraint stress. RNA sequencing (RNA‐seq) was performed to identify oligodendrocytes and stress‐associated molecules in the nucleus accumbens (NAcc) of both drug users and cocaine‐treated animals. Further, we studied which cell subtypes in the brain express CRYAB. The effects of stress hormones and cocaine on CRYAB expression were evaluated in vitro in human oligodendrocytes. CRYAB is upregulated in the NAcc of both cocaine‐treated animals and drug users. CRYAB levels in the NAcc of mice increased during CPP development but decreased following stress‐induced relapse. Interestingly, CRYAB is expressed in oligodendrocytes in the NAcc of mice. Extracellular CRYAB levels are regulated by cocaine and stress hormone treatments in oligodendrocyte cultures. Dopamine levels in the NAcc and CPP development of CPP are significantly increased by cocaine in CRYAB knockout (KO) mice. Further, we demonstrated that CRYAB binds to the excitatory amino acid transporter 2 (EAAT2) in the NAcc of mice treated with cocaine. We suggest that oligodendrocyte‐derived CRYAB regulates dopamine neurotransmission and stress‐evoked cocaine reward behaviour via the modulation of EAAT2 in the NAcc.

## Graphical abstract

This study examined CRYAB, an oligodendrocyte‐enriched protein, and its role in dopamine and glutamate regulation in the NAcc via EAAT2. Cocaine and stress altered CRYAB expression, affecting cocaine‐induced CPP. Restraint stress triggered CPP relapse, with CRYAB playing a role in this mechanism, suggesting its involvement in stress‐induced drug reinforcement (created in https://BioRender.com).

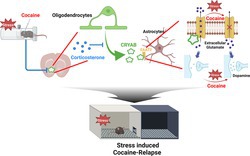

## 1 Introduction

Stress is an environmental risk factor for both drug abuse development and relapse. Both human and animal studies have revealed a correlation between chronic stress and the behavioural and neurobiological characteristics of drug addiction [1]. Several neurotransmission systems have previously been suggested as targets of stress‐induced drug abuse, including endocannabinoids, dopamine, corticotropin‐releasing factor and glutamate [2, 3]. However, the precise molecular mechanisms underlying acute stress‐evoked drug reward behaviour, as assessed using the conditioned place preference (CPP) paradigm, have yet to be completely elucidated.

The nucleus accumbens (NAcc) is a key brain region implicated in the mechanisms of addiction and reward processing [4, 5]. However, existing research on addiction and reward has predominantly concentrated on targeting neurons within brain regions linked to addiction, including the NAcc [6, 7]. Further, other cell types besides neurons may also be associated with drug addiction and relapse [8]. For example, the role of glial cells in drug addiction has been previously suggested, whereas astrocytes have been indicated to regulate drug reward behaviour via modulation of neurotransmitter uptake [9]. Cytokines and growth factors released from microglia have also been implicated in neuronal adaptation to drugs [10]. Indeed, several studies have revealed the involvement of oligodendrocytes in addiction behaviours [11, 12] or stress [13, 14, 15]. Heroin self‐administration can upregulate the expression of myelin‐associated genes [11], such as myelin basic protein (MBP), whereas methamphetamine decreases expression [16]. Research has indicated that Sox10 expressed in oligodendrocytes of the PFC plays a critical role in regulating motivated behaviours [11]. Alterations in neurotrophic factors secretion from oligodendrocyte‐precursor cells (OPC), which is associated with Sox10, was also suggested as a possible mechanism underlying the role of oligodendrocytes in drug addiction [11]. OPC is also related with the homeostatic response to stress exposure [17]. The structural abnormalities and redox imbalance observed in chronic stress animal models may be related with myelination deficits in major depressive disorder. However, other oligodendroglial molecular changes involved in the stress‐induced reinstatement of drug addiction remain largely unclear.

Crystallin alpha B (CRYAB) is a heat shock protein that exerts a protective role against multiple sclerosis in animal models [18]. This chaperone protein is predominantly mainly in oligodendrocytes and astrocytes in the central nervous system [19]. Its physiological role is associated with the inhibition of apoptosis and inflammatory response in the myelin sheath [18]; however, its association with drug addiction remains unclear.

Herein, we identified that CRYAB in the nucleus accumbens (NAcc) is associated with drug use in humans and sensitization to cocaine in animals. We further analysed CRYAB expression levels in the NAcc of mice at each stage of CPP development, extinction and relapse. The effects of stress hormones and cocaine on the CRYAB levels in human oligodendrocytes were also investigated. Additionally, we tested whether CRYAB‐knockout (KO) mice were vulnerable to the development of cocaine‐induced CPP and further studied the molecular mechanisms underlying the possible role of CRYAB in cocaine rewarding behaviour. Extracellular dopamine levels induced by cocaine were also measured in the NAcc of CRYAB‐KO mice. Furthermore, we identified CRYAB‐binding molecules using liquid chromatography with tandem mass spectrometry (LC‐MS/MS) and immunoprecipitation, which may regulate dopaminergic neurotransmission.

## 2 Materials and Methods

### 2.1 Animals

This study was conducted in strict accordance with the recommendations of the Guide for the Care and Use of Laboratory Animals, and all experiments were approved by the Guidelines for the Care and Use of Animals (Animal Care Committee of Chungbuk National University, Cheongju, Korea [CBNUA‐2027‐22‐02]). Male C57BL/6 N mice and Sprague–Dawley (SD) rats at 7 weeks of age were sourced from Daehan Biolink (Eumsung, Korea). Male CRYAB KO mice with a C57BL/6N genetic background were developed using CRISPR/Cas9‐based methods (Figures S1–S2) (Macrogen Co. Ltd., Seoul, Republic of Korea) [20]. Animal rooms were maintained at a constant temperature (21°C–24°C) and relative humidity (40%–60%) with a 12‐h light/dark cycle (lights on 08:00–20:00). Animals were provided with a solid diet and tap water ad libitum. Animal experiments were initiated after a 1‐week acclimatization period and were performed during the light cycle (09:00–18:00). The animals were euthanized by CO_2_ suffocation within 30 min of completion of the experiment. For immunohistochemistry (IHC), mouse hearts were perfused with PBS and 4% paraformaldehyde, whereas the brains were extracted from the skull. Perfused brains were incubated in 4% paraformaldehyde at 4°C for 72 h and subsequently dehydrated by sequential incubation in 10%, 20% and 30% sucrose solutions for 24 h each. For molecular analysis (qPCR, western blotting, etc.), brains were extracted from skulls immediately after euthanasia, whereas brain matrix was used to isolate specific regions (e.g., the NAcc and CPu), and samples were immediately stored at −80°C.

### 2.2 Materials

Cocaine hydrochloride (HCl) was purchased from MacFarlan Smith Ltd. (Edinburgh, United Kingdom). Corticosterone and false‐positive neurotransmitters (FFN102) were purchased from Sigma‐Aldrich (St. Louis, MO, United Kingdom).

### 2.3 Locomotor Activity on Cocaine‐Induced Sensitization

The rats (*n* = 20) were injected intraperitoneally (i.p.) with saline or 15 mg/kg cocaine HCl for 5 days, followed by two drug‐free days. Locomotor activity was measured on Day 8 (ENV520; Med Associates Inc., Fairfax, VT, United States), both 60 min before and after the administration of 15 mg/kg cocaine HCl.

### 2.4 Quantitative Real‐Time Polymerase Chain Reaction (qPCR)

For mRNA quantification, total RNA was extracted from the rat brains using a commercial total RNA extraction kit (iNtRON Biotechnology, Seongnam, Republic of Korea). Complementary DNA (cDNA) was synthesized from total isolated RNA using the SuperScript III First‐Strand Synthesis Kit (Invitrogen, Carlsbad, CA, United States). qPCR was performed using an iCycler iQ5 Real‐Time Detection System (Bio‐Rad, Hercules, CA, United States) using SYBR GreenER qPCR SuperMix Universal (Invitrogen, Carlsbad, CA, United States) and primers (Table S1). The average value was calculated after conducting two technical replicates. The results were normalized to the levels of glyceraldehyde 3‐phosphate dehydrogenase (GAPDH) levels and quantified relative to the expression in the control samples. For relative quantification, the 2^−ΔΔCt^ formula was used:

$$\begin{array}{c} \text{ΔΔCt}=\left({\mathrm{Ct}}_{\text{target}}-{\mathrm{Ct}}_{\text{GAPDH}}\right)\;\text{experimental sample}-\\ \;\left({\mathrm{Ct}}_{\text{target}}-{\mathrm{Ct}}_{\text{GAPDH}}\right)\;\text{control sample}. \end{array}$$

### 2.5 RNA Sequencing (RNA‐Seq) of Human Postmortem Brain Samples and RNA‐Seq Data Processing and Differentially Expressed Gene Analysis

The Institutional Review Board (IRB) of the Uniformed Services University of Health Sciences, Bethesda, MD, granted ethical approval to the Stanley Brain Collection. The collection period was between 1998 and 2004, all human participants were deceased and all specimens were deidentified and simply numbered; consequently, the IRB determined that approval was not required. Consent to donate the specimens was obtained from the next of kin and confirmed by two people who signed a form verifying the fact. Subsequently, the next of kin were contacted and interviewed to obtain further information on the deceased. RNA samples were extracted from the NAcc of 79 individuals from array collections (AC). RNA‐seq experiments were performed using a Psomagen (Rockville, MD, United States). The detailed method is described in Method S1.

### 2.6 CPP Test on Restraint Stress‐Induced Cocaine Relapse

To elucidate the effect of stress on cocaine‐induced reward behaviour in mice, we examined whether restraint stress increased CPP reinstatement. The CPP test was performed according to a previously described method, with minor modifications [21]. The detailed method is presented in Method S2.

### 2.7 Induction of Restraint Stress in Mice

The induction of restraint stress in mice was performed in the reinstatement phase of mice undergoing the CPP test or normal C57BL/6 N mice. In brief, mice were placed in a well‐ventilated 50‐mL conical tube (polystyrene/high‐density polyethylene, 30.0‐mm external dimension and 116.7‐mm height; SPL Life Sciences, Pocheon‐si, Republic of Korea) for 30 min, with quartered wooden chopsticks placed in the conical tube to fill the space between the tube cap and mouse [22, 23].

### 2.8 Immunohistochemistry/Immunofluorescence (IHC/IF)

The brains were sampled immediately following the developmental phase of the CPP schedule (Figure 2A). All procedures were similar to those used in a previous study [21]. The detailed methods are described in Method S3.

### 2.9 Culture of Human Primary Oligodendrocytes

Primary human oligodendrocytes (36055‐22, Celprogen, Torrance, CA, United States) were seeded into 24‐well plates (E36055‐22‐24 Well, Celprogen) at a density of 1 × 10^5^ cells/well. These cells were cultured in complete growth media (M36055‐22S, Celprogen) under 5% CO_2_ with a high atmospheric humidity (90%–95%) at 37°C.

### 2.10 ELISA of CRYAB in the Oligodendrocyte Culture Media

Primary oligodendrocytes were cultured for 24 h in control and cocaine‐treated media (1 or 10 μM). The medium was subsequently replaced with vehicle‐ or corticosterone‐treated medium (200 ng/mL). After 1 h, the medium was collected and centrifuged at 14 000 rpm and 4°C for 10 min. Subsequently, only the supernatant was collected. All procedures used the CRYAB ELISA kit (IMMUNOSET® αB‐Crystallin ELISA development set, Enzo Biochem Inc., Fabringdale, NY, United States), following the manufacturer's guidelines.

### 2.11 Ex Vivo Imaging

Wild‐type (WT) and CRYAB‐KO mice were acutely injected with saline or cocaine (i.p., 10 mg/kg). Thirty minutes following the injection, the striatum, including the NAcc, was sliced coronally at 1‐mm intervals using a brain matrix (68 713, RWD Life Science, CA, United States). The detailed method is described in Method S4.

### 2.12 Stereotaxic Surgery, Microdialysis and Dopamine Measurement

Stereotaxic surgery was performed as previously described [21]. The stereotaxic coordinates of the NAcc were as follows: 0.5 mm anterior to the bregma, 1.3 mm lateral to the sagittal suture, and 4.2 mm ventral to the brain surface. A microdialysis probe with a 1‐mm active membrane (FX‐I‐6‐01; Eicom, Kyoto, Japan) was inserted into the guide cannula (AG‐6; Eicom). Artificial perfusion of cerebrospinal fluid (ACSF; 125 mM NaCl, 26 mM NaHCO_3_, 3 mM KCl, 1.6 mM CaCl_2_, 1.5 mM MgSO_4_, 1.25 mM NaH_2_PO_4_ and 10 mM glucose; pH 7.4) was performed for 1.5 h before treatment with cocaine (10 mg/kg, i.p.). The dialysate was collected every 10 min at a rate of 2.0 μL/min (approximately 20 μL) and added to 5 μL of 0.1‐M perchloric acid. The dopamine levels of all dialysate samples were analysed using HPLC, according to a previous report [21].

### 2.13 Coimmunoprecipitation (Co‐IP) Assay

Immunoprecipitation assays were performed using a commercial kit, in accordance with the manufacturer's instructions (Pierce Classic Magnetic IP/Co‐IP kit, WH331624, Thermo Fisher Scientific, Waltham, MA, United States). Control or cocaine‐injected mouse brain samples (NAcc) were mixed with CRYAB antibody (1:1000; ab13496, Abcam) and incubated overnight at 4°C. Protein A/G beads were subsequently added to the reaction mixture. The CRYAB‐binding proteins were eluted using an elution buffer.

### 2.14 Investigation of CRYAB Binding Molecules

To identify the target molecule that directly interacts with CRYAB during cocaine addiction, LC‐MS/MS analysis was performed using co‐IP samples. Brain samples were eluted using the coimmunoprecipitation method, whereas only CRYAB‐binding proteins were purified. The samples were then analysed using a Q Exative Plus Hybrid Quadrupole Orbitra Mass Spectrometer (Figure S4). Genes were identified using Proteome Discoverer 2.4 software. In addition, g:Profiler (https://biit.cs.ut.ee/gprofiler/gost) for GO, KEGG, and REAC, and GeneMANIA (https://genemania.org/) were used for functional annotation and target gene selection, respectively (Figure S5).

### 2.15 Western Blotting

Brain samples were prepared and used for western blotting, as previously described [21, 24]. All procedures subsequent were similar to those used in a previous study [21]. The detailed methods are described in Method S5.

### 2.16 CPP Test on CRYAB KO Mice

To elucidate the effect of CRYAB on cocaine‐induced reward behaviour in mice, we examined the effect of cocaine‐induced CPP in WT and CRYAB‐KO mice. The CPP test was conducted under unbiased and counterbalanced conditions. The detailed procedure is illustrated in Figure 6A.

### 2.17 Data Analysis

Data are presented as the means ± standard error (SE). Data were analysed using Student's *t* test, one‐ and two‐way analysis of variance (ANOVA) and two‐way repeated measures (RM) ANOVA, followed by the Holm‐Sidak post hoc *t* test using SigmaPlot 12 software (Systat Software, Chicago, IL, United States).

## 3 Results

### 3.1 Cocaine Increased the Locomotor Activity

**FIGURE 1:**
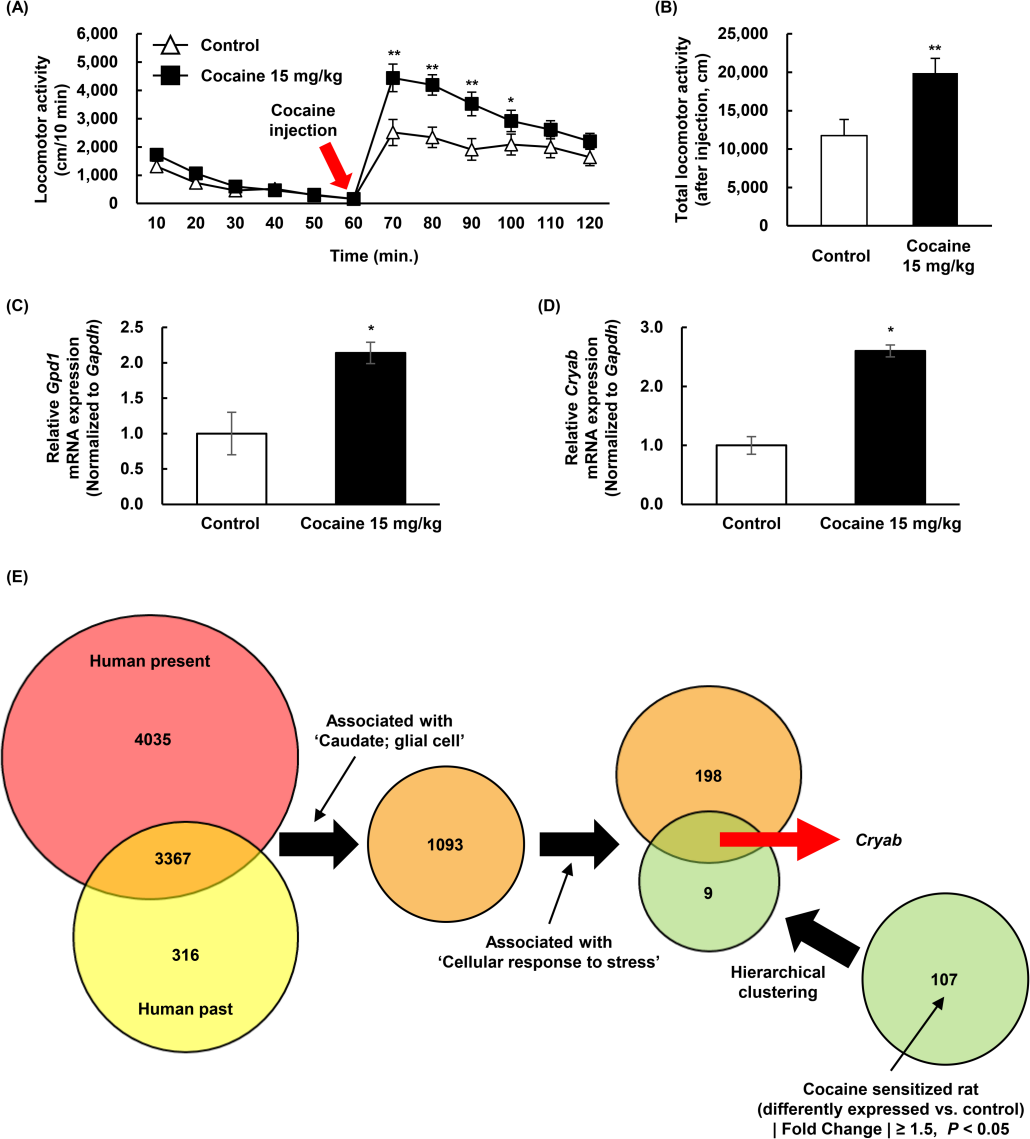
Quantification of gene expression levels in rats exhibiting cocaine‐induced sensitization of locomotor activity and comparison with gene expression in human. (A, B) Rats are injected with either saline‐control or cocaine 15 mg/kg (i.p.). (A) Locomotor activity is measured at 10‐min intervals for 120 min. Data are expressed as the mean distance travelled ± SE (*n* = 20) and are analysed using two‐way repeated measure ANOVA followed by the Holm–Sidak post hoc *t* test (**p < 0.01 and *p < 0.05 vs. control [at the same time point]). (B) Total locomotor activity is measured for 120 min. Data are expressed as the mean total distance travelled ± SE (*n* = 20) and are analysed using Student's *t* test (**p < 0.01 vs. control). (C, D) The mRNA expression level of the (C) *Gpd1* and (D) *Cryab* detected in the microarray is confirmed using qPCR with each specific primer. The expression of each gene is normalized to the relative amplification of each *Gapdh*. Data are expressed as the mean ± SE (*n* = 4, Student's *t* test, *p < 0.05 vs. control). (E) Differently expressed genes both in the human past and present drug user groups are anatomically and functionally categorized. The differently expressed genes in the cocaine‐sensitized rats are detected via hierarchical clustering. The genes detected in the two different species are compared to identify the target proteins associated with cocaine relapse. All samples are extracted from the NAcc of the human and rat brains.

To verify the cocaine‐induced sensitization in rats, we performed a locomotor test. This test revealed remarkable hyperlocomotion in cocaine‐pretreated (15 mg/kg) rats (Figure 1A: *n* = 20; *F*_*drugs*_[1, 38] = 10.891, *p* = 0.002; *F*_*time*_[11 418] = 43.02, *p* < 0.001; *F*_*drugs*×*time*_[11, 418] = 4.56, *p* < 0.001) (Figure 1B: *n* = 20; *p* = 0.0069), indicating a high sensitivity to cocaine.

### 3.2 CRYAB Was Identified in Cocaine‐Sensitized Rats and Human Drug Users

Omics analysis was conducted to identify commonly altered genes in the brains, specifically the NAcc region, of rats sensitized to cocaine and human subjects with a history of substance abuse. Significant differentially expressed genes were detected in the NAcc of cocaine‐induced sensitization rats (**|**Fold Change**|** ≥ 1.5, *p* < 0.05, vs. control) using the DNA microarray method. The fold change was calculated based on the expressed gene probes between the comparison samples (cocaine‐induced sensitization [cocaine pretreated] and cocaine desensitization [cocaine non‐pretreated] groups). Target gene candidates were reselected via hierarchical clustering (*n* = 10 genes) (Table S3 and Figure S6). qRT‐PCR was further performed to quantify mRNA levels. Only the mRNA levels of *Gpd1* (Glycerol‐3‐phosphate dehydrogenase 1) and *Cryab* were significantly higher in the cocaine group than in the control group (Figure 1C, *n* = 4; *p* = 0.0139) (Figure 1D: *n* = 4; *p* = 0.0012) (Figure S7). The genes detected in the cocaine‐treated rat NAcc were cross‐analysed with the differentially expressed genes in the NAcc of past human users (*n* = 3683 genes) and current human users (*n* = 7403 genes) and compared with those of control nonusers (Figure 1E). First, concurrently expressed genes in both past and present drug users (*n* = 3367 genes) were recategorized via anatomical and functional annotation. The 1093 genes expressed in human caudate glial cells were subsequently categorized, from which 198 genes associated with ‘cellular response to stress’ were filtered out. Finally, genes selected from the two species were compared. Only *Cryab* appeared at the intersection between the human drug users and cocaine‐sensitized rats (Figure 1E). Thus, we conclude that CRYAB may be a target molecule that plays a key role in oligodendrocytes and is associated with cocaine relapse.

### 3.3 Restraint Stress Provoked Cocaine Relapse in CPP

**FIGURE 2:**
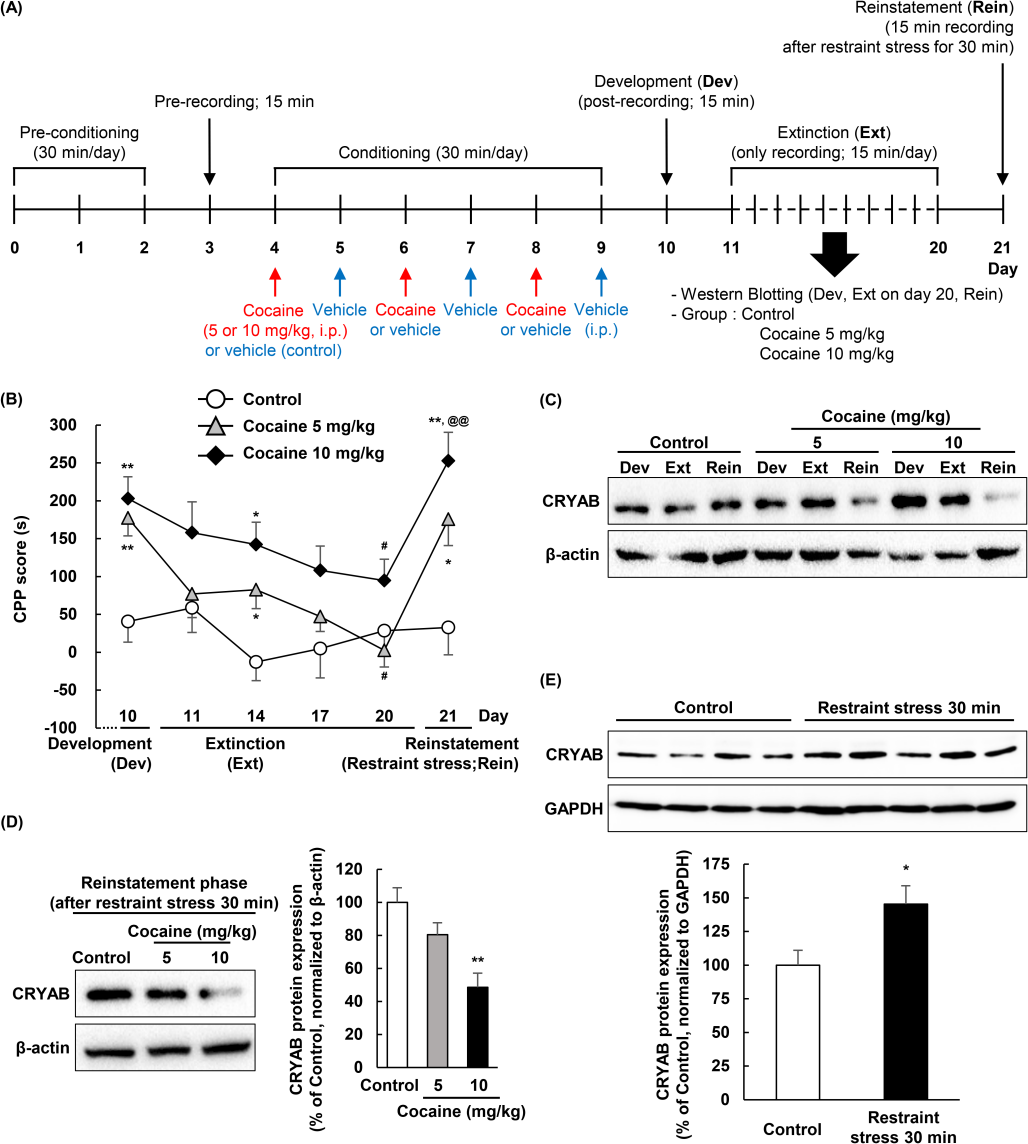
Restraint stress induces relapse in cocaine‐conditioned place preference and alters the expression pattern of CRYAB. (A) Diagram illustrating the timeline of procedures on cocaine‐induced CPP development phase, extinction phase, and restraint stress‐induced reinstatement phase in C57BL/6N wild‐type mice. Postrecording was performed at three different stages of addiction: development phase, on each day of the extinction phase and immediately after restraint stress was applied in the reinstatement phase. The time that each mouse spent in each compartment is recorded and used to determine the preference for each compartment for 15 min. On Day 21, 1 day after the last extinction day, restraint stress was applied for 30 min by restraining the mouse in a 50‐mL conical tube, and then, the CPP was immediately tested. (B) Before starting the extinction, the mice were developed by cocaine (5 or 10 mg/kg, i.p.) in the CPP test. Restraint stress‐induced CPP score(s) is measured after the extinction phase. Data are expressed as the mean ± SE (*n* = 9–18) and are analysed using two‐way repeated measure ANOVA followed by the Holm–Sidak post hoc *t* test (**p < 0.01 and *p < 0.05 vs. control [at the same day point], ^#^ *p* < 0.05 vs. Day 10 for each group [each development phase] and ^@@^ *p* < 0.01 vs. Day 20 of each group [last extinction date for each group]). (C–E) The protein expression in NAcc of CRYAB is detected by Western blotting with specific antibodies and normalized to β‐actin. (C) The CRYAB expression is presented on each phase (*n* = 1). (D) Data are expressed as the mean ± SE (*n* = 4) and are analysed using one‐way ANOVA followed by the Holm–Sidak post hoc *t* test (**p < 0.01 vs. control). (E) Data are expressed as the mean ± SE (*n* = 9 and 10 for the control and cocaine 10 mg/kg groups, respectively) and are analysed using Student's *t* test (*p < 0.05 vs. control). NAcc, nucleus accumbens.

Restraint stress during the conditioning phase inhibited the development of drug (methamphetamine) abuse‐induced CPP (Figure S8). This result is consistent with those of a previous report [25]. Subsequently, to reveal how stress during the reinstatement phase impacts cocaine‐relapse behaviour (Figure 2A), we compared the CPP score immediately following restraint stress in the reinstatement phase and those of the phase of development and extinction (Figure 2B: *F*_*drugs*_[2, 47] = 12.166, *p* < 0.001; *F*_*phase*_[5159] = 5.413, *p* < 0.001; *F*_*drugs*×*phase*_ [10 159] = 1.271, *p* = 0.251). In the development phase, the CPP scores in both the 5‐ and 10‐mg/kg‐injected groups were higher than those in the control group (*n* = 16, 16 and 18 for the control, cocaine 5 mg/kg and cocaine 10 mg/kg groups, respectively; **p < 0.01). In the extinction phase, the CPP scores gradually decreased over time, and by Day 20 (the last day of the extinction phase), both the 5‐ and 10‐mg/kg‐injected groups had significantly lower CPP scores than those in each development phase (*n* = 14, 9 and 11 for the control, cocaine 5 mg/kg and cocaine 10 mg/kg groups, respectively; ^#^ *p* < 0.05). During the reinstatement phase, restraint stress was applied instead of cocaine treatment to confirm whether restraint stress could trigger cocaine relapse. Both cocaine groups showed significantly higher CPP scores based on the restraint stress compared to the control (*n* = 12, 12 and 14 for the control, cocaine 5 mg/kg and cocaine 10 mg/kg groups, respectively; **p < 0.01 and *p = 0.018); however, only the cocaine 10‐mg/kg‐injected group showed significantly higher CPP scores in the reinstatement phase (Day 21) than those on the last extinction day (Day 20) (^@@^ *p* = 0.004). These results indicate that restraint stress in the reinstatement phase evoked cocaine‐induced CPP relapse, in a manner dependent on the cocaine dose in the development phase.

### 3.4 CRYAB Expression Decreased During Stress‐Induced Relapse

CRYAB expression in the NAcc was quantified using Western blotting at each phase of the CPP schedule (Figure 2A). The relative expression of CRYAB appeared to increase following the administration of 10 mg/kg cocaine during the developmental phase (Figure 2C). At the end of the extinction phase (Day 20), the CRYAB levels increased following treatment with 10 mg/kg cocaine (Figure 2C). However, in the reinstatement phase (Day 21), CRYAB expression was significantly decreased in the cocaine 10 mg/kg group during stress‐induced CPP relapse (Figure 2D: *n* = 4; *F*[2, 9] = 10.088, *p* = 0.005). These findings indicate that CRYAB expression was increased and maintained during the development and extinction phases; however, it was reduced by restraint stress during the cocaine relapse phase.

### 3.5 Restraint Stress Elevated the CRYAB Expression

To determine the acute effect of stress on CRYAB expression without drug treatment, restraint stress was applied to naïve mice. Subsequently, we performed Western blotting on freshly obtained NAcc brain tissue, which showed that a single restraint stress session increased CRYAB expression in the NAcc (*n* = 9 and 10 for the control and restraint stress 30 min groups, respectively; *p* = 0.02) (Figure 2E). These results indicate that stress‐induced alterations in CRYAB expression are dependent on cocaine treatment.

### 3.6 Cocaine Increased the CRYAB Expression in NAcc Oligodendrocytes

**FIGURE 3:**
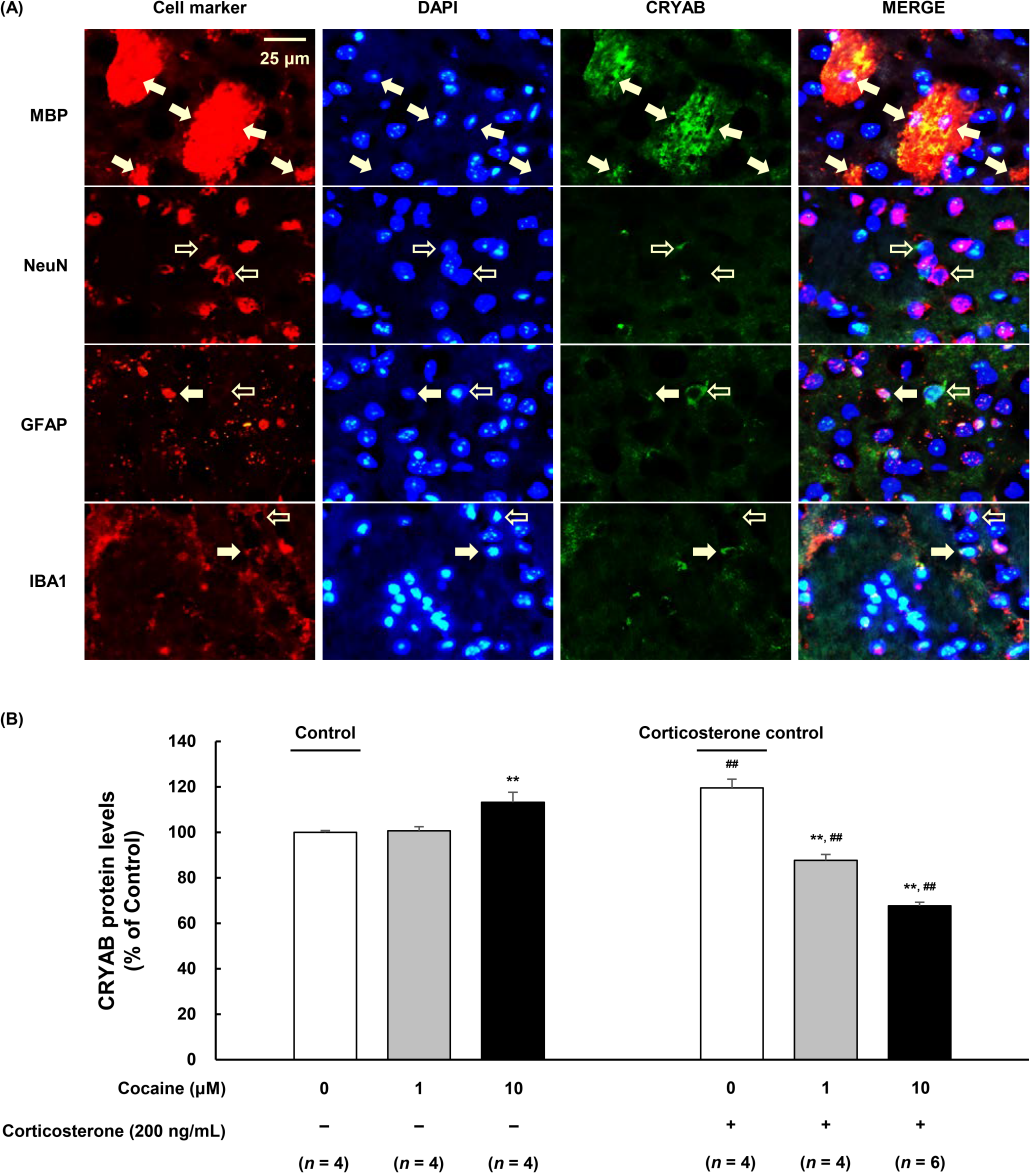
CRYAB‐expressing cell types in the cocaine‐administered mice NAcc and reduced Extracellular CRYAB levels in oligodendrocytes in stress‐induced relapse. (A) The mice are injected with cocaine (5 mg/kg [i.p.]). The mice brain sections (10‐μm‐thick) are reacted with anti‐CRYAB (green) and antieach brain cell marker (red). MBP, NeuN, GFAP and IBA1 are markers of oligodendrocytes, neurons, astrocytes and microglia, respectively. Closed arrows indicate the colocalization of each marker and CRYAB. Open arrows indicate the non‐colocalization of each marker and CRYAB. Original magnification is 200X. The scale bar is 25 μm. (B) The CRYAB protein levels are measured in culture media of primary human oligodendrocytes by using ELISA kit. Primary human oligodendrocytes are cultured in control or cocaine‐treated media (1 or 10 μM) for 24 h, followed by treatment with corticosterone (200 ng/mL) for 1 h. Data are expressed as the mean ± SE (*n* = 4 for each cocaine‐treated group without corticosterone, corticosterone control and 1‐μM cocaine‐treated group with corticosterone, respectively; *n* = 6 for 10 μM cocaine‐treated group with corticosterone) and are analysed using two‐way repeated measure ANOVA followed by the Holm–Sidak post hoc *t* test (**p < 0.01 vs. each control, ^##^ *p* < 0.01 vs. each cocaine at the same treatment dose). NAcc, nucleus accumbens.

To determine the specific cell type expressing CRYAB, IHC/IF was performed for various protein markers in brain sections of cocaine‐pretreated mouse striataum. CRYAB expression was not observed in neuronal cells (anti‐NeuN), but this protein was partially expressed in astrocytes (anti‐GFAP) and microglia (anti‐IBA1). However, CRYAB was abundantly expressed in most oligodendrocytes (anti‐MBP) (Figure 3A). These findings indicate that CRYAB may be expressed in oligodendrocytes in the NAcc of mice.

### 3.7 Extracellular CRYAB Concentration Was Altered by Corticosterone and Cocaine Treatment in Oligodendrocytes

As described above, CRYAB was predominantly expressed in the oligodendrocytes of the NAcc (Figure 3A). CRYAB is secreted extracellularly by exosomes [26, 27, 28, 29]. To investigate whether CRYAB acts via an extracellular pathway, CRYAB levels were measured in the culture media of human oligodendrocytes using an ELISA kit (Figure 3B: *F*_*cocaine*_[2, 20] = 28.859, *p* < 0.001; *F*_*corticosterone*_ [1, 20] = 35.177, *p* < 0.001; *F*_*cocaine*×*corticosteron*_[2, 20] = 75.911, *p* < 0.001). This analysis showed that the protein level of CRYAB increased with cocaine treatment (10 μM, 24 h) (*p* = 0.009 vs. control). Acute corticosterone treatment (200 ng/mL, 1 h) increased the extracellular protein levels of CRYAB in the culture media (*p* < 0.01 vs. each control). However, the protein levels of CRYAB decreased in the corticosterone‐administered group (200 ng/mL) following exposure to cocaine for 24 h, both at 1 and 10 μM (**p < 0.01 vs. each control and ^##^ *p* < 0.01 vs. each cocaine at the same treatment dose of cocaine). These results were consistent with the CRYAB expression levels observed in the CPP experiments (Figure 2C–E).

### 3.8 CRYAB Deficiency Decreased the Dopamine Transporter (DAT) Availability

**FIGURE 4:**
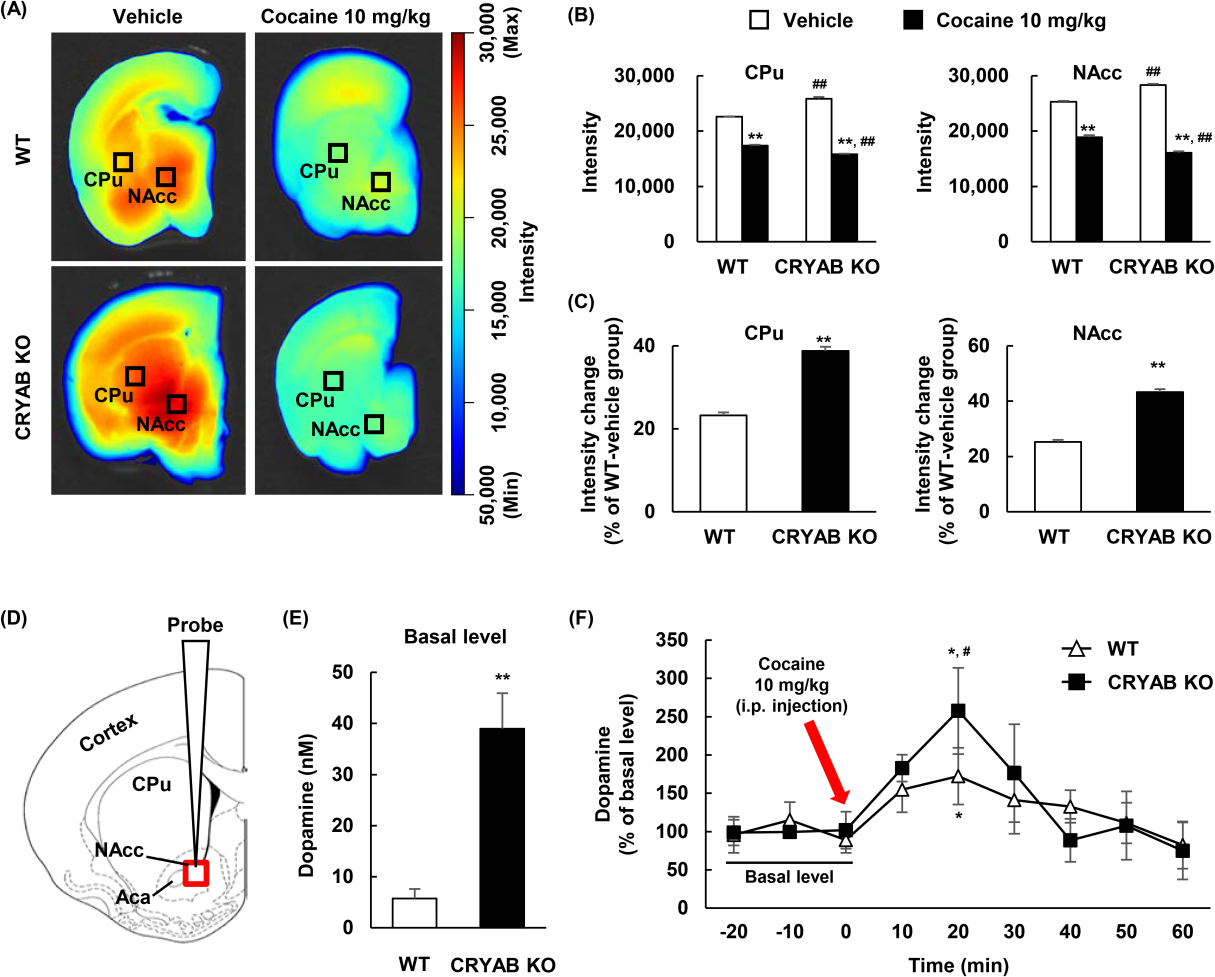
Dopamine transporter availability and dopamine levels in CRYAB KO mice. (A–C) DAT availability is measured in the striatal brain slices of WT and CRYAB KO mice. DAT availability is visualized using a false fluorescent neurotransmitter (FFN102, 500 μM). Black square is a marker on the area of caudal putamen (CPu) and NAcc in the brain slice for calculating the fluorescence. (B) Data are expressed as the intensity mean ± SE (*n* = 6 and 4 for the intensity on CPu and NAcc groups, respectively) and are analysed using two‐way repeated measure ANOVA followed by the Holm–Sidak post hoc *t* test (**p < 0.01 vs. each vehicle control, ^##^ *p* < 0.01 vs. each WT‐pair). (C) Data are expressed as the intensity change (% of WT‐vehicle group) mean ± SE (*n* = 6 and 4 for the intensity on CPu and NAcc groups, respectively) and are analysed using Student's *t* test (**p < 0.01 vs. each WT group). (D–F) The dopamine levels are measured in the mice NAcc via microdialysis probe. (D) Regions where the microdialysis probe is inserted. (E) The basal levels of dopamine are measured. Data are expressed as the mean ± SE (*n* = 4) and are analysed using Student's *t* test (**p < 0.01 vs. WT mice). (F) Cocaine (10 mg/kg, i.p.)‐induced dopamine levels (% of basal level) are measured. Data are expressed as the mean ± SE (*n* = 4) and are analysed using two‐way repeated measure ANOVA followed by the Holm–Sidak post hoc *t* test (*p < 0.05 vs. each basal level [at 0 min], ^#^ *p* < 0.05 vs. WT group at the same time). NAcc, nucleus accumbens.

DAT availability was measured using FFN102 (500 μM), a known substrate of DAT that competitively binds to DAT, replacing dopamine [30]. Therefore, a decreased FFN102 intensity indicates low DAT availability, indicating that the dopamine levels are altered in the extracellular area. ROI analyses were performed to calculate the fluorescence intensity in the NAcc and caudal putamen (CPu) (Figure 4A). The intensity levels of FFN102 in both the CPu and NAcc of both WT and CRYAB KO mice were significantly reduced in the cocaine‐treated group compared with the vehicle group and were noticeably higher in CRYAB KO mice than in WT mice (Figure 4B_CPu_: *n* = 6; *F*_*gene*_[1, 20] = 21.927, *p* < 0.001; *F*_*drug*_[1, 20] = 1753.976, *p* < 0.001;*F*_*gene*×*drug*_[1, 20] = 172.631, *p* < 0.001; Figure 4B_NAcc_: *n* = 4; *F*_*gene*_[1, 12] = 0.137, *p* = 0.718; *F*_*drug*_[1, 12] = 1261.713, *p* < 0.001; *F*_*gene*×*drug*_[1, 12] = 125.109, *p* < 0.001). Subsequently, the intensity change ([*F*_*control*_ – *F*_*cocaine*_]/*F*_*control*_ × 100 [%]) was calculated and compared between WT and CRYAB KO mice. CRYAB KO mice showed a more dramatic decrease in FFN102 intensity in both the CPu and NAcc (Figure 4C_CPu_: *n* = 6; *p* < 0.01; Figure 4C_NAcc_: *n* = 4; *p* < 0.01). We confirmed the interaction between CRYAB and DAT using GeneMANIA (https://genemania.org/) (Figure S9A), for which the results indicated that CRYAB may be involved in regulating the magnitude of changes in DAT availability induced by cocaine.

### 3.9 Extracellular Dopamine Level Was Augmented in CRYAB KO Mice

We performed an in vivo test using the microdialysis method to collect and analyse dopamine in the NAcc of mice (Figure 4D). Spontaneous dopamine levels were significantly higher in CRYAB‐KO than in WT mice (*n* = 4, *p* = 0.003) (Figure 4E). During cocaine treatment, the dopamine levels as a percentage of each basal level increased in both the WT and CRYAB KO groups, particularly when the knockout of the CRYAB gene was more significant than in the WT group (Figure 4F: *n* = 4; *F*_*gene*_[1, 6] = 0.219, *p* = 0.657; *F*_*time*_[8, 48] = 3.46, *p* = 0.003; *F*_*gene*×*time*_[8, 48] = 0.857, *p* = 0.558). These results, including the DAT availability results, indicated that CRYAB is involved in cocaine‐induced changes in dopamine levels in the NAcc of mice.

### 3.10 EAAT2 Emerged as the CRYAB Binding Molecule

**FIGURE 5:**
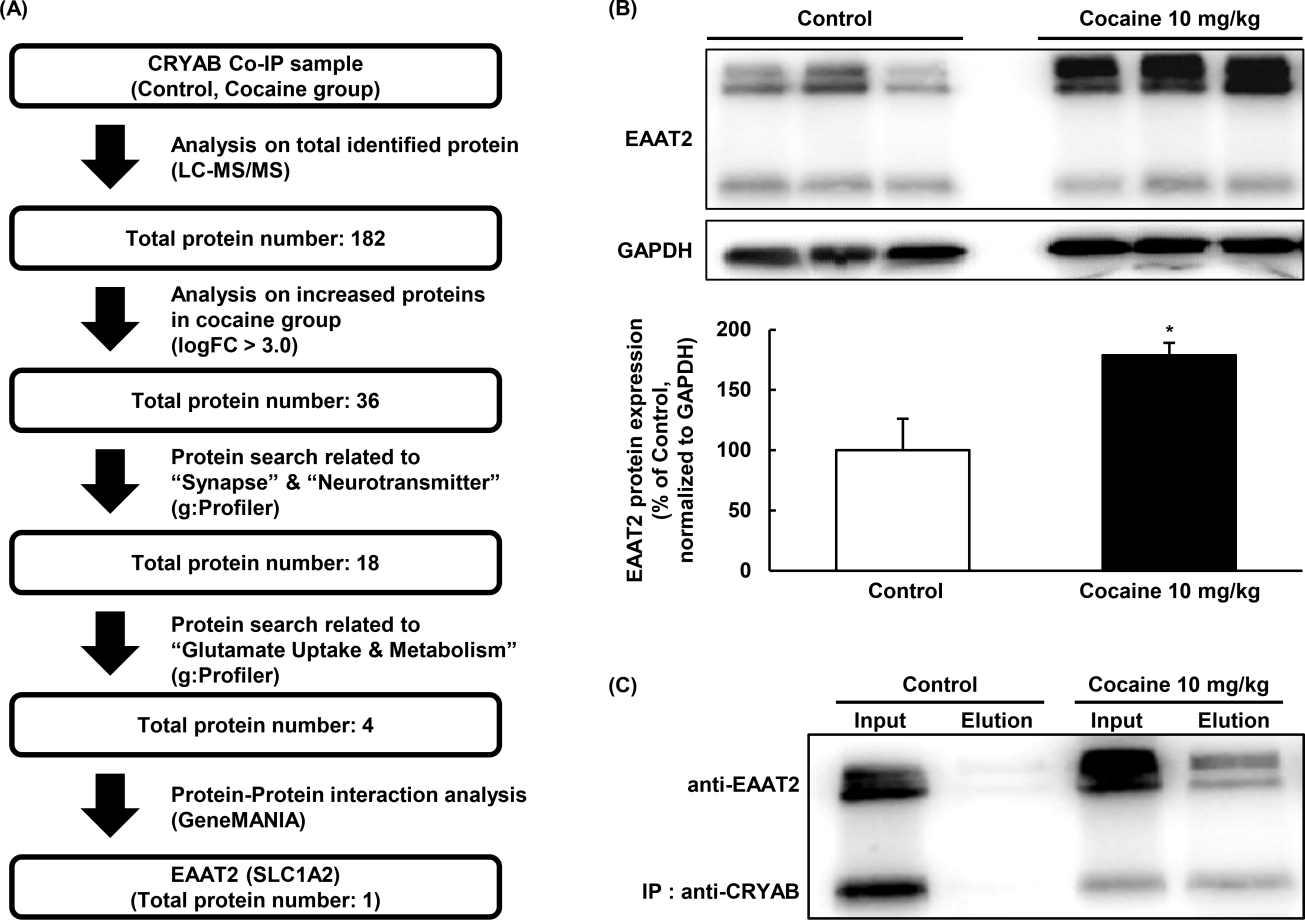
Schematic diagram and detection of the target protein binding to CRYAB. (A) Each step followed for detecting the target protein is briefly described. (B) The protein expression of EAAT2 is detected using Western blotting with specific antibodies and normalized to GAPDH in the NAcc of mice. Data are expressed as the means ± SE (*n* = 3 and 6 for the control and cocaine 10 mg/kg groups, respectively) and are analysed using Student's *t* test (*p < 0.05 vs. control). (C) Co‐IP and Western blotting are performed to reveal whether the binding of EAAT2 to CRYAB in cocaine‐treated mice NAcc. The samples of mice NAcc are used for Co‐IP with CRYAB. After Co‐IP, the elution samples are detected using Western blotting with EAAT2 antibody.

In the LC‐MS/MS study, we used CRYAB‐binding proteins from co‐IP samples with mouse brain samples (NAcc) treated with 10 mg/kg control or cocaine, which yielded 182 proteins. Among these proteins, we identified several upregulated proteins in the cocaine‐treated group (*n* = 36, fold‐change > 3.0, vs. control, *p* < 0.05). Several proteins were reselected and enriched in categories related to synaptic function and neurotransmitter uptake (*n* = 18) and were subsequently reselected in categories related to glutamate uptake and metabolism (*n* = 4) using g:Profiler (Figures 5A and S5). Functional and physical interactions between these proteins and CRYAB were investigated using GeneMANIA (https://genemania.org/) (Figure S9B). Our results showed that glutamate transporter 1 (GLT‐1; EAAT2) was the sole protein directly associated with CRYAB (*n* = 1). Therefore, EAAT2, which may be associated with the modulatory role of CRYAB in cocaine addiction, was selected as the target protein.

### 3.11 CRYAB Binding to EAAT2 Increased With Cocaine Treatment

The expression of EAAT2 was analysed in the mouse striatum (including the NAcc) and compared between the control and cocaine‐treated groups (i.p., 10 mg/kg). Protein samples were prepared immediately following CPP development and at the end of the postrecording period. EAAT2 expression was significantly increased (approximately 1.78‐fold change) in the cocaine‐treated group (Figure 5B: *n* = 3 and 6 for the control and cocaine 10 mg/kg groups, respectively; *p* = 0.011). Co‐IP was subsequently performed to verify whether CRYAB bound to EAAT2 in the mouse striatum. EAAT2 normally showed a double band, at approximately 65 and 180 kDa [31]. The control group showed either no band or only a single vague band (approximately 180 kDa) in the Western blot. In contrast, double bands (at approximately 65 and 180 kDa) appeared in the cocaine‐treated group (Figure 5C).

### 3.12 CRYAB Deficiency Elevated the CPP

**FIGURE 6:**
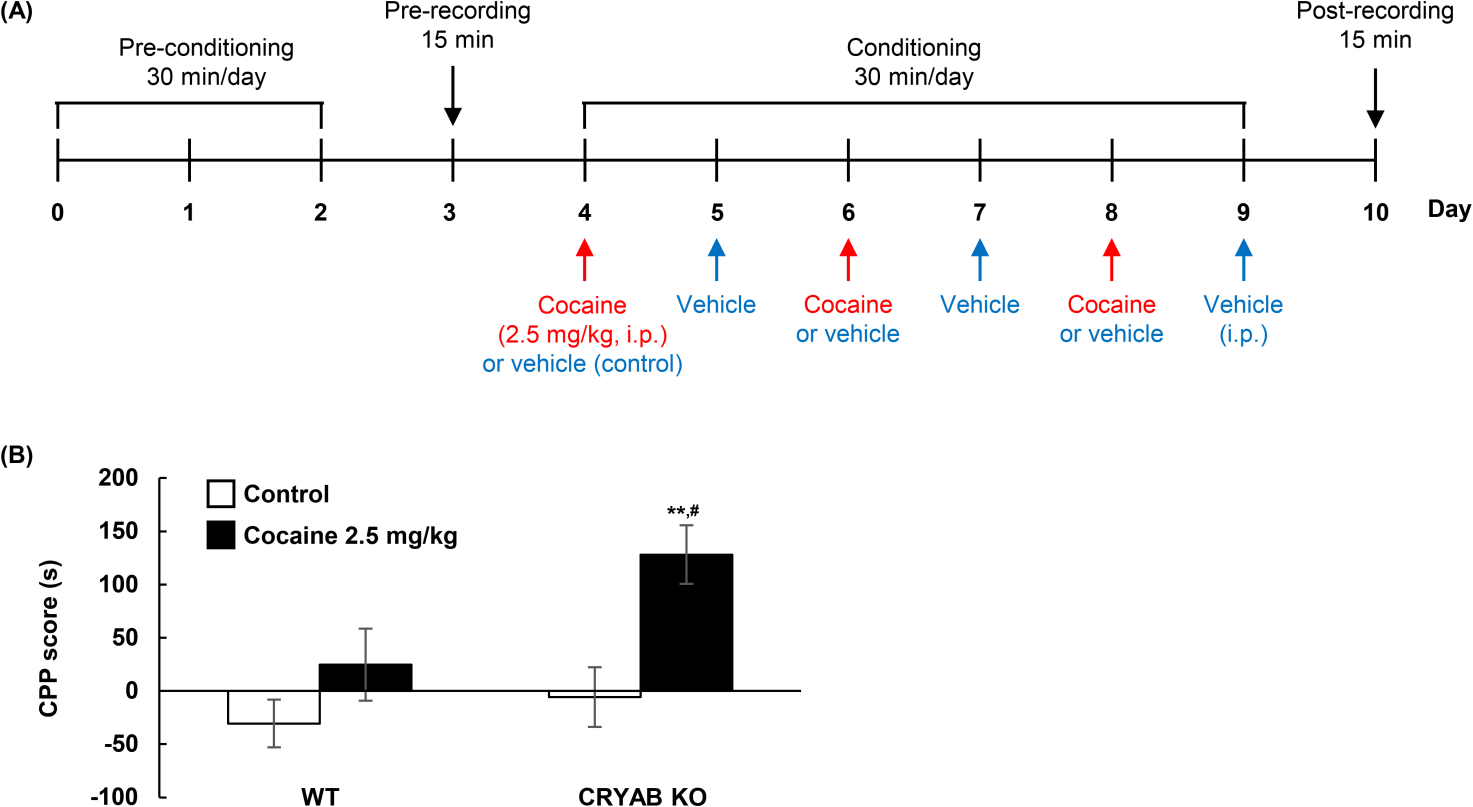
Conditioned place preference induced by cocaine was increased in Cryab knockout mice. (A) Diagrams illustrating the timeline of cocaine‐induced CPP procedures in wild‐type and CRYAB KO mice. (B) Cocaine‐induced CPP score are measured using the CPP test. Data are expressed as the mean ± SE (*n* = 12, 11, 9 and 11 for WT‐control, WT‐cocaine, CRYAB KO‐control and CRYAB KO‐cocaine, respectively) and are analysed using two‐way repeated measure ANOVA followed by the Holm–Sidak post hoc *t* test (**p < 0.01 vs. CRYAB KO‐control, ^#^ *p* < 0.05 vs. WT‐cocaine 2.5 mg/kg). NAcc, nucleus accumbens.

We verified that the administration of cocaine (5 and 10 mg/kg) induced CPP development (Figure 2B), whereas a high dose (10 mg/kg) of cocaine induced a remarkable increase in dopamine levels in the NAcc of CRYAB KO mice in vivo (Figure 4F). Therefore, we investigated whether a low dose of cocaine (2.5 mg/kg), which does not induce CPP in WT mice, could induce CPP in CRYAB KO mice, to avoid ceiling effects of high doses of cocaine (Figure 6A,B: *n* = 12, 11, 9 and 11 for the WT‐control, WT‐cocaine, CRTAB KO‐control and CRYAB KO‐cocaine groups, respectively; *F*_*gene*_[1, 39] = 5.355, *p* = 0.026; *F*_*drug*_[1, 39] = 11.68, *p* = 0.001; *F*_*gene*×*drug*_[1, 39] = 2.002, *p* = 165). We found no difference between the control and cocaine‐treated mice in the WT group (*p* = 0.149); however, there was a significant increase in the CPP score between the control and cocaine 2.5 mg/kg‐treated mice in the CRYAB KO group (**p = 0.002). Moreover, comparing the cocaine‐treated groups, CRYAB KO mice showed a much higher CPP score than WT mice (^##^ *p* = 0.011). CRYAB deficiency resulted in the development of CPP, even at a low dose (2.5 mg/kg) of cocaine, whereas this effect was not observed in WT mice, indicating that CRYAB may be involved in the development of reward behaviour in cocaine.

## 4 Discussion

Numerous studies have previously suggested that glial cells respond to stress, consequently contributing to neuronal adaptation [32]. Glial cells may play a role in neuronal homeostasis and are associated with stress‐evoked drug reward behaviour [33, 34, 35]. However, the role of oligodendrocytes in the modulation of stress on the conditional response to cocaine remains unclear. To identify the underlying mechanisms of glial cells in stress‐evoked rewards, we sought to identify target molecules associated with glial cell function. Our investigation demonstrated that oligodendrocytes express CRYAB, which is upregulated in the NAcc of animal models locomotor‐sensitized by repeated cocaine treatment. In these cocaine‐sensitized animals, differentially expressed genes in the NAcc were cross‐analysed with human data from past and present drug users. Interestingly, only the expression of the *Cryab* gene was elevated compared with that in the control group in both the human and rat gene pools. This finding suggests that CRYAB may be highly related to reward function.

CRYAB is also known as a stress‐resistant molecule, and stress‐induced upregulation of CRYAB has been previously investigated [36, 37]. Furthermore, stress‐induced relapse of substance use, including of alcohol, heroin and cocaine, has been reported [38, 39]. However, the relationship between CRYAB and stress‐induced drug relapse has not been studied. First, we examined whether CPP relapse was affected by acute restraint stress following the cessation of CPP development due to intermittent cocaine administration. Cocaine administration induced CPP in a dose‐dependent manner, which was gradually extinguished. To induce relapse, mice were exposed to acute restraint stress after the extinction of cocaine‐induced CPP. Exposure to stress after extinction induced CPP in all cocaine dose groups. However, stress exposure during the conditioning phase may further contribute to the development of CPP induced by cocaine [25]. Similarly, our data revealed that restraint stress during the conditioning phase inhibited CPP development (Figure S8). These results indicate that restraint stress could inhibit the development of CPP but enhance memory reconsolidation during relapse. Second, we demonstrated that CRYAB expression in the NAcc was upregulated by cocaine in a dose‐dependent manner during development. Furthermore, CRYAB expression was significantly reduced in mice in relapse status compared to that in the vehicle group in a cocaine dose‐dependent manner. In contrast, CRYAB expression in the NAcc was significantly increased by acute restraint stress in noncocaine‐pretreated animals. In summary, restraint stress augmented CRYAB expression.

Conversely, in cocaine‐addicted animals, stress reduces CRYAB expression in the NAcc, suggesting that stress‐induced CRYAB expression depends on the drug experience in the CPP paradigm. According to previous reports [40, 41], cocaine induces epigenetic alterations in the genome that may regulate neuronal adaptation during drug abuse. Glucocorticoids regulate the transcription of stress response genes [42]. CRYAB contains glucocorticoid response elements in its promoter region that bind to several transcription factors (Figure S9). It has also been reported that cocaine and amphetamine regulate the expression levels of transcription factors related to stress response [43]. As such, cocaine pretreatment may alter the epigenetic regulation of CRYAB, consequently reducing its stress‐evoked expression in mice undergoing cocaine relapse.

To clarify the role of CRYAB in cocaine‐induced reward behaviour, we demonstrated that CRYAB‐KO mice developed CPP following exposure to a low dose of cocaine (2.5 mg/kg), which did not induce CPP in WT mice. This behavioural phenotype in CRYAB KO mice may be attributed to an elevation in extracellular dopamine levels in the NAcc, induced, at least in part, by cocaine. This finding suggests a novel regulatory mechanism for CRYAB in dopaminergic neurotransmission. CRYAB was abundantly expressed in oligodendrocytes, defined as MBP positive cells of the corpus callosum, in the white matter (Figure S10). In our study, CRYAB expression increased in MBP‐positive cells in the NAcc after cocaine administration. We further observed that CRYAB regulates neuronal functions such as dopamine release. Therefore, we speculated that extracellular CRYAB may play a role in the interaction between oligodendrocytes and dopaminergic neurons. We further examined whether extracellular CRYAB expression in oligodendrocytes was affected by cocaine or corticosterone. In cultured oligodendrocytes, CRYAB levels in culture media increased following a single treatment with cocaine or corticosterone but were inhibited by posttreatment with corticosterone after being cultured in cocaine‐containing media. CRYAB is a secreted molecule that interacts with exosomes [44, 45]. These results show the same pattern as the change in CRYAB expression at different CPP stages, indicating that CRYAB modulates cocaine reward via an extracellular function.

Furthermore, we demonstrated that CRYAB binds to EAAT2 and that this binding is augmented by cocaine treatment. EAAT2 is a glutamate transporter expressed in neurons and glial cells [46]. Drug abuse induces the dysregulation of glutamate transport, which is associated with drug‐seeking behaviour [47]. Further, cocaine evokes glutamate release in the reinstatement phase, which can contribute to drug‐seeking behaviour [48, 49]. CRYAB is a chaperone molecule that directly modulates dopaminergic neuronal function by binding to the target molecule, which was shown to be EAAT2 in a coimmunoprecipitation experiment. Furthermore, in the present study, cocaine increased the expression of CRYAB and EAAT2 in the NAcc. Consequently, we assumed that CRYAB expression responds to the cocaine‐induced glutamate increase and may further play a homeostatic function to preserve the normal functioning of glutamatergic neurotransmission through EAAT2. Dysregulated glutamate neurotransmission has been associated with altered dopaminergic neuronal function in the NAcc. This interaction between glutamate and the dopamine system in the NAcc may be modulated by CRYAB, resulting in upregulation of dopamine levels.

## 5 Conclusions

Taken together, the results of this study suggest that oligodendrocyte‐derived CRYAB plays a role in stress‐induced cocaine relapse by regulating the effects of EAAT2 on both dopaminergic and glutamatergic neuronal function.

## Author Contributions

Conceptualization: Hye Jin Cha and Jaesuk Yun. Data curation: Sun Mi Gu and Daejin Park. Formal analysis: Sun Mi Gu, Daejin Park, Sowoon Seo, Sanghyeon Kim and Maree J. Webste. Funding acquisition: Jin Tae Hong, Hye Jin Cha and Jaesuk Yun. Investigation: Sun Mi Gu, Daejin Park, Young Eun Kim, Sowoon Seo, Sanghyeon Kim, Heejong Eom and Dohyun Lee. Methodology: Sanghyeon Kim, Maree J. Webster, Heejong Eom, Dohyun Lee, Jin Tae Hong and Sang‐Bae Han. Project administration: Hye Jin Cha and Jaesuk Yun. Resources: Sanghyeon Kim, Maree J. Webster, Dohyun Lee, Jin Tae Hong, Sang‐Bae Han, Hye Jin Cha and Jaesuk Yun. Software: Sanghyeon Kim and Maree J. Webster. Supervision: Hye Jin Cha and Jaesuk Yun. Visualization: Sun Mi Gu, Daejin Park and Sowoon Seo. Writing – original draft: Sun Mi Gu and Daejin Park. Writing – review and editing: Hye Jin Cha and Jaesuk Yun.

## Ethics Statement

This study was conducted in strict accordance with the recommendations of the Guide for the Care and Use of Laboratory Animals, and all experiments were approved by the Guidelines for the Care and Use of Animals (Animal Care Committee of Chungbuk National University, Cheongju, Korea [CBNUA‐2027‐22‐02]). In RNA‐seq, the Institutional Review Board (IRB) of the Uniformed Services University of the Health Sciences, Bethesda, MD, granted ethical approval for the Stanley Brain Collection. As the collection period was between 1998 and 2004 and the human participants deceased, the IRB determined that approval was not required, as all specimens were deidentified and simply numbered. Consent to donate the specimens was obtained from next of kin and witnessed by two people who signed a form verifying the fact. Subsequently, the next of kin was contacted and interviewed to obtain further information about the deceased.

## Conflicts of Interest

The authors declare no conflicts of interest.

## Supporting information


**Table S1** Primer sequences used for qRT‐PCR analysis of cocaine‐sensitized rats.
**Table S2** Demographic and clinical variables for human samples used in this study.
**Table S3** Up‐ or down‐regulated genes in the nucleus accumbens of cocaine‐sensitized rats.
**Figure S1** Altered Transcription site of exon in CRYAB KO mice. (A) Four types of gRNA in the ATG start exon target site. (B) Sequence of designed gRNAs. gRNA, guide RNA
**Figure S2** Materials and PCR conditions for CRISPR/Cas9 experiments. (A) Components and methods for the synthesis of sgRNA and Cas9 protein. (B) Components and PCR conditions for the confirmation of CRYAB cleaved form.
**Figure S3** Human RNA‐seq analysis process. Quality control of the raw FASTQ files, read mapping, and read quantification were conducted as previously described^1^ with the following modifications. To enhance accuracy and obtain comprehensive gene expression profiles, we used the GRCh38 reference genome assembly in HISAT2 to align our RNA‐seq reads. Additionally, to identify and remove outlier samples from our RNA‐seq dataset, we employed a clustering‐based approach. This method resulted in the exclusion of one sample from the data analysis. The quality of raw data from all samples is initially checked using FastQC (https://www.bioinformatics.babraham.ac.uk/projects/fastqc/) and passed. Sequencing reads are mapped to the human reference genome, GRCh38, using HISAT2 (version 2.2.1)^3^. Counting of the mapped reads is performed using HTseq‐count (subprogram of HTseq, version 2.0.1)^4^ with the GRCh38 annotation file, and no strand‐specific and intersection‐nonempty options. Next, we identified and adjusted for confounding variables in the normalized read count data using surrogate variable analysis (SVA package, version 3.50.0)^5^. Then, we conducted a clustering analysis on the adjusted read count data to detect outlier samples and the samples were excluded from the downstream analysis. The final differentially expressed genes (DEGs) analysis included RNA‐Seq data from the NAcc of the 15 past users, 14 present users, and 49 controls. To identify the DEGs in the NAcc between drug users and controls, we compared the read counts of genes from both groups using a generalized linear model with surrogate covariates in EdgeR software (version 3.31.5)^6^. A false discovery rate (FDR) of < 0.05 is considered significant. For functional annotation of the DEGs, the Enrichment of Kyoto Encyclopedia of Genes and Genomes (KEGG) pathways enriched in these genes are identified using gProfiler^7^. The FDR is computed using the Benjamini–Hochberg method to correct the error rate of multiple testing. An FDR of < 0.05 is considered significant. DEG, differentially expressed gene; FDR, false discovery rate, HISAT2, hierarchical indexing for spliced alignment of transcripts; HT‐seq, high‐throughput sequencing; NAcc, nucleus accumbens.
**Figure S4** Equipment, reagents, and detailed conditions used for LC‐MS/MS analysis.
**Figure S5** Omics analysis to find proteins associated with CRYAB. After analysis on increased proteins (logFC > 3.0) in cocaine group on LC‐MS/MS data from CRYAB Co‐IP sample (control and cocaine‐treated group), analysis on GO, KEGG, and REAC using by g:Profiler (https://biit.cs.ut.ee/gprofiler/gost) were performed to find proteins associated with (A) synapse & neurotransmitter and (B) glutamate uptake & metabolism.
**Figure S6** Hierarchical clustering of the detected genes from the DNA microarray assay. The cocaine‐sensitized rats and the control group are sacrificed on the 8th day, after the locomotor test, and the NAcc was dissected for analysis. The microarray process is executed according to the manufacturer’s protocol (GeneChipTH Whole Transcript PLUS reagent kit, 902280). We exported the results of gene‐level RMA analysis and performed DEG analysis. The FDR is controlled by adjusting the p‐value using the Benjamini–Hochberg algorithm. For a DEG set, hierarchical cluster analysis is performed using complete linkage and the Euclidean distance as a measure of similarity. Gene enrichment and functional annotation analysis for the significant probe list are performed using DAVID (http://david.abcc.ncifcrf.gov/home.jsp). All data analyses and visualization of the differentially expressed genes are conducted using R 3.1.2 (www.r‐project.org). DEG, differentially expressed gene; FDR, false discovery rate; NAcc, nucleus accumbens; RMA, robust multi‐array average.
**Figure S7** mRNA expression level of the target genes upon cocaine administration. (A‐G) The mRNA expression level of the target genes detected in the microarray is confirmed using qPCR with each specific primer. The expression of each gene is normalized to the relative amplification of each *Gapdh*. Data are expressed as the mean ± S.E. (*n* = 4, Student’s t‐test). The p‐values for each gene are as follows: (A) 0.9365, (B) 0.9485, (C) 0.9695, (D) 0.8060, (E) 0.4385, (F) 0.3365, and (G) 0.3035.
**Figure S8** Effect of restrain stress on methamphetamine (1 mg/kg)‐induced CPP development. Restraint stress is applied for 30 min by restraining the mouse in a 50‐mL conical tube. The conditioning phase is then immediately commenced. Data are expressed as the mean ± S.E. (*n* = 4) and are analyzed using Student’s t‐test (*p = 0.027 vs. control).
**Figure S9** Analysis on gene–gene interaction with CRYAB. Interaction map showing the reference‐based correlation between CRYAB (CRYAB coding gene) and (A) SLC1A2 [glutamate transporter 1 (EAAT2) coding gene] or (B) SLC6A3 [dopamine transporter (DAT) coding gene] using GeneMANIA (http://genemania.org/).
**Figure S10** *Cryab* promotor region includes GRE binding sites. (A) The ‐2 kb sites of the *Cryab* promotor include a CpG island, where is a 5’‐CG‐3’ rich region of DNA. The CpG island region includes GR motifs (red square). (B) GR‐binding motifs in the site at –5,000 bp from the initiation of the *Cryab*. (C) GR motif‐binding genes enhancing *Cryab* transcription in humans. CG, cytosine and guanine; CpG, 5’—C—phosphate—G—3’; GR, glucocorticoid receptor.
**Figure S11** CRYAB‐expressing cell types in the cocaine‐administered mice corpus callosum. The mice are injected with cocaine 5 mg/kg (i.p.). The mice brain sections (10‐μm‐thick) are reacted with anti‐CRYAB (green) and anti‐MBP, anti‐NeuN, anti‐GFAP, and anti‐IBA1 (brain cell markers; red). MBP, NeuN, GFAP, and IBA1 are markers of oligodendrocytes, neurons, astrocytes, and microglia, respectively. Closed arrows indicate the co‐localization of each marker and CRYAB. Open arrows indicate the non‐co‐localization of each marker and CRYAB. The original magnification is ×200. The scale bar is 25 μm. CC, corpus callosum; LS, lateral septal nucleus.

## Data Availability

The data that support the findings of this study are available on request from the corresponding author. The data are not publicly available due to privacy or ethical restrictions.
