# Supplementary material for: Crystallin Alpha B Inhibits Cocaine‐Induced Conditioned Place Preference via the Modulation of Dopaminergic Neurotransmission
Source: Addict Biol. 2025 Mar 17;30(3):e70028. doi: 10.1111/adb.70028 (PMC11912016; doi:10.1111/adb.70028)
Supplement: Supplementary file 1 — Table S1 Primer sequences used for qRT‐PCR analysis of cocaine‐sensitized rats. Table S2 Demographic and clinical variables for human samples used in this study. Table S3 Up‐ or down‐regulated genes in the nucleus accumbens of cocaine‐sensitized rats. Figure S1 Altered Transcription site of exon in CRYAB KO mice. (A) Four types of gRNA in the ATG start exon target site. (B) Sequence of designed gRNAs. gRNA, guide RNA Figure S2 Materials and PCR conditions for CRISPR/Cas9 experiments. (A) Components and methods for the synthesis of sgRNA and Cas9 protein. (B) Components and PCR conditions for the confirmation of CRYAB cleaved form. Figure S3 Human RNA‐seq analysis process. Quality control of the raw FASTQ files, read mapping, and read quantification were conducted as previously described1 with the following modifications. To enhance accuracy and obtain comprehensive gene expression profiles, we used the GRCh38 reference genome assembly in HISAT2 to align our RNA‐seq reads. Additionally, to identify and remove outlier samples from our RNA‐seq dataset, we employed a clustering‐based approach. This method resulted in the exclusion of one sample from the data analysis. The quality of raw data from all samples is initially checked using FastQC (https://www.bioinformatics.babraham.ac.uk/projects/fastqc/) and passed. Sequencing reads are mapped to the human reference genome, GRCh38, using HISAT2 (version 2.2.1)3. Counting of the mapped reads is performed using HTseq‐count (subprogram of HTseq, version 2.0.1)4 with the GRCh38 annotation file, and no strand‐specific and intersection‐nonempty options. Next, we identified and adjusted for confounding variables in the normalized read count data using surrogate variable analysis (SVA package, version 3.50.0)5. Then, we conducted a clustering analysis on the adjusted read count data to detect outlier samples and the samples were excluded from the downstream analysis. The final differentially expressed genes (DEGs) analysis include [file ADB-30-e70028-s001.docx]

**Supplementary Information**

**Supporting information Methods S1** RNA sequencing.

In brief, cDNA libraries were prepared from 0.5 µg of RNA per sample, using the Illumina TruSeq RNA Library commercial kit (Illumina Inc., San Diego, CA, USA), following the manufacturer’s instructions. Sequencing libraries were created and sequenced on an Illumina platform.  Base calling and demultiplexing were performed using bcl2fastq (bcl2fastq-1.8.4; Illumina). Quality control of the raw FASTQ files, read mapping, and read quantification were conducted as previously described^1^ with the following modifications: To enhance the accuracy and obtain comprehensive gene expression profiles, we used the GRCh38 reference genome assembly in HISAT2 to align the RNA-seq reads. Further, we employed a clustering-based approach to identify and remove outlier samples from the RNA-seq dataset, resulting in the exclusion of one sample from the data analysis. On average, 56 million paired-end reads were generated per sample. Approximately 50 million reads were mapped to annotated gene features using the GRCh38.95.gtf annotation file, enabling the quantification of the expression levels of 57,189 genes. Raw FASTQ files can be requested from the SNCID database (<https://sncid.stanleyresearch.org/>),^2^ which will provide an ID and password for downloading files via SFTP at sftp://bidas.kaist.ac.kr/. The entire procedure is illustrated in the supporting information Figure S3. The sample included the following: 1) individuals with past drug use, not using drugs at the time of death (past users; *n* = 15), 2) individuals using drugs at the time of death (present users; *n* = 14), and 3) individuals who had never used drugs (non-users; *n* = 50) (Supporting Information Table S2). Omics analysis on both cocaine-sensitized rats and human drug users were performed based on the analysis of the human protein atlas (HPA) and gene ontology (GO), associated with 'Caudate; glial cell' and 'Cellular response to stress' using g:Profiler (https://biit.cs.ut.ee/gprofiler/gost).

**Supporting information Methods S2** CPP test on restraint stress-induced cocaine relapse.

The CPP test comprised four phases (Figure 2A), as follows i) The pre-conditioning phase (days 0 to 2): Mice were allowed free access to all compartments of the apparatus for 30 min/day for three days. ii) The pre-recording phase (day 3): The time spent by mice in each compartment was recorded for 15 min. Mice that spent significantly more time in one compartment than in the other (70% vs. 30%) were excluded. iii) The conditioning phase (days 4 to 9): Conditioning was conducted over six days, with one session per day. On days 4, 6, and 8, mice were treated with either saline (i.p.) or cocaine (5 or 10 mg/kg/day, i.p.), and placed in a white compartment for 30 min. On days 5, 7, and 9, the mice were treated with saline (i.p.), and placed in a black compartment for 30 min. iv) Development (post-recording; day 11): The time spent by the mice in each compartment was recorded for 15 min. v) Extinction (days 11–20): The time spent by the mice in each compartment was recorded for 15 min. The graph of the CPP scores for the extinction phase is presented only for days 11, 14, 17, and 20. vi) Reinstatement phase (day 21): The method for reinstatement induction by restraint stress is presented in the Materials and Methods section 2.7. The time spent by the mice in each compartment was recorded 15 min following reinstatement. The CPP score for each phase was calculated as follows:

(1) Development phase: Change in the time spent in the drug-pair chamber during the post-recording (development) and pre-recording phases.

(2) Extinction phase: Change in time spent in the drug pair chamber during each extinction and pre-recording phase.

(3) Reinstatement phase: Change in the time spent in the drug pair chamber during the reinstatement and pre-recording phases.

**Supporting information Methods S3** Immunohistochemistry/Immunofluorescence (IHC/IF)

Coronal slices (10-μm-thick) of the brain, including the striatum and NAcc, were used for this experiment. Brain sections were incubated in anti-MBP (1:1,000; ab40390, Abcam, Cambridge, UK), anti-ionized calcium binding adaptor molecule 1 (IBA1; 1:1,000; ab48004, Abcam), anti-glial fibrillary acidic protein (GFAP; 1:1,000; ab53554, Abcam), anti-neuronal nuclear protein (NeuN; 1:1,000; ab177487, Abcam), and anti-CRYAB (1:1,000; ab13496, Abcam) at 4°C overnight, as well as the appropriate Alexa Fluor 488- and 594-linked secondary antibodies and DAPI (300 nM; Sigma-Aldrich). Brain sections were evaluated under a light microscope (Axio Imager.A2, Carl Zeiss, Oberkochen, Germany; × 200).

**Supporting information Methods S4** *ex vivo* imaging

Brain slices were washed twice with phosphate-buffered saline (PBS) for 5 min (× 2), and fixed in 4% paraformaldehyde for 30 min. After washing three times (10 min each) with PBS, FFN102 solution (500 μM; Sigma-Aldrich) was added to treat the sliced tissue in a 24-well cell culture dish for 1 h on a slow shaker without light. Green fluorescence was subsequently measured using an *in vivo* optical imager (VISQUE^®^ in Vivo Smart-LF, ViewWorks, Anyang, Republic of Korea). The region of interest (ROI) intensity was analyzed in the caudate putamen (CPu) and NAcc areas using CleVue™ (ViewWorks).

**Supporting information Methods S5** Western blotting

The following primary antibodies were used for Western blotting: anti-excitatory amino acid transporter 2 (EAAT2; 1:1,000; ab205248; Abcam), anti-CRYAB (1:1,000; ab13496; Abcam), and anti-GAPDH (1:1,000; 14C10; Cell Signaling Technology, Waltham, MA, USA). Blots were incubated with appropriate horseradish peroxidase-linked secondary antibodies and visualized using a FUSION Solo S chemiluminescence detection system (Vilber Lourmat, Collégien, France) with ECL solution (Millipore, Billerica, MA, USA). The relative density of the protein bands was confirmed using ImageJ software ( National Institutes of Health, Bethesda, MD, USA).

**Supporting information Table S1** Primer sequences used for qRT-PCR analysis of cocaine-sensitized rats.

| **Gene name** | **Forward primer (5'→3')** | **Reverse primer (5'→3')** | **Product**  **Size (bp)** |
| --- | --- | --- | --- |
| *Fam111a* | ACACCAGGAACCAAAGACCAC | TCACACCCAAGGTGACCAGA | 93 |
| *Slc17a7* | CGAGTCACCTGCACTACACC | ATGAGCTTGGCGCTTTCTCC | 84 |
| *Nr4a2* | CAGTATGGGTCCTCGCCTCA | GCTGTATTCTCCCGAAGAGTGG | 75 |
| *Olr1194* | CTCCCCCTCATGAAGCTTTCC | GTGACGACAATGTTGAACCCAG | 86 |
| *Adcyap1* | AGCTTCGGCAAACAAGTCCC | GCGCTGGAATCACAACCAGA | 108 |
| *Cryab* | TTCTACCTTCGGCCACCCTC | GCTTCACGTCCAGGTTCACA | 109 |
| *Gpd1* | TGCACAGCATCCTCCAACAC | GCAGCAGATGAACTCACCCA | 107 |
| *Olr522* | ATATGTGCTCACTGTCCTGGGG | TCAATGCAAGACAAGTGGGTGA | 108 |
| *Cnksr2* | CCGGCTTTCCTCCACAGAGA | ACTGGCTGTCTTGCGAATGG | 106 |
| *Gapdh* | AGTTCAACGGCACAGTCAAG | TACTCAGCACCAGCATCACC | 118 |

**Supporting information Table S2** Demographic and clinical variables for human samples used in this study.

|  | **Non-users**  **(N=50)** | **Past users**  **(N=15)** | **Present users**  **(N=14)** |
| --- | --- | --- | --- |
| **Age** | 47±8.3 | 43±7.5 | 38±8.5^**^ |
| **Sex (M/F)** | 31/19 | 8/7 | 11/3 |
| **Brain pH** | 6.5±0.31 | 6.5±0.22 | 6.5±0.30 |
| **PMI** | 32±16 | 35±13 | 30±14 |
| **RIN** | 6.9±1.2 | 6.9±1.3 | 7.3±1.1 |
| **Diagnosis (U/B/S)** | 27/9/13 | 0/9/16^***^ | 1/4/9^**^ |

Data are expressed as the mean ± S.D. and analyzed using pairwise comparison after ANOVA test using by R package (^**^*p* < 0.01 and ^***^*p* < 0.001 vs. non-user group). PMI, post-mortem interval; RIN, RNA integrity number; U, unaffected control; B, bipolar disorder; S, schizophrenia.

**Supporting information Table S3** Up- or down-regulated genes in the nucleus accumbens of cocaine-sensitized rats.

| **Gene name** | **Gene Description** | **Fold Change**  **(Cocaine/Control)** | ***P*-value** |
| --- | --- | --- | --- |
| *Fam111a* | Family with sequence similarity 111, member A | 2.0644 | 0.0005 |
| *Slc17a7* | Solute carrier family 17 (vesicular glutamate transporter), member 7 | 1.87504 | 0.0008 |
| *Nr4a2* | Nuclear receptor subfamily 4, group A, member 2 | 1.86903 | 7.79E-05 |
| *Olr1194* | Olfactory receptor 1194 | 1.83597 | 0.0296 |
| *Adcyap1* | Adenylate cyclase activating polypeptide 1 | 1.7808 | 1.85E-05 |
| *Cryab* | Crystallin, alpha B | 1.66709 | 0.0063 |
| *Gpd1* | Glycerol-3-phosphate dehydrogenase 1 (soluble) | 1.52819 | 0.0012 |
| *Mir421* | MicroRNA mir-421 | -1.8516 | 0.0102 |
| *Olr522* | Olfactory receptor 522 | -1.5165 | 0.0044 |
| *Cnksr2* | Connector enhancer of kinase suppressor of Ras 2 | -1.5066 | 0.017 |


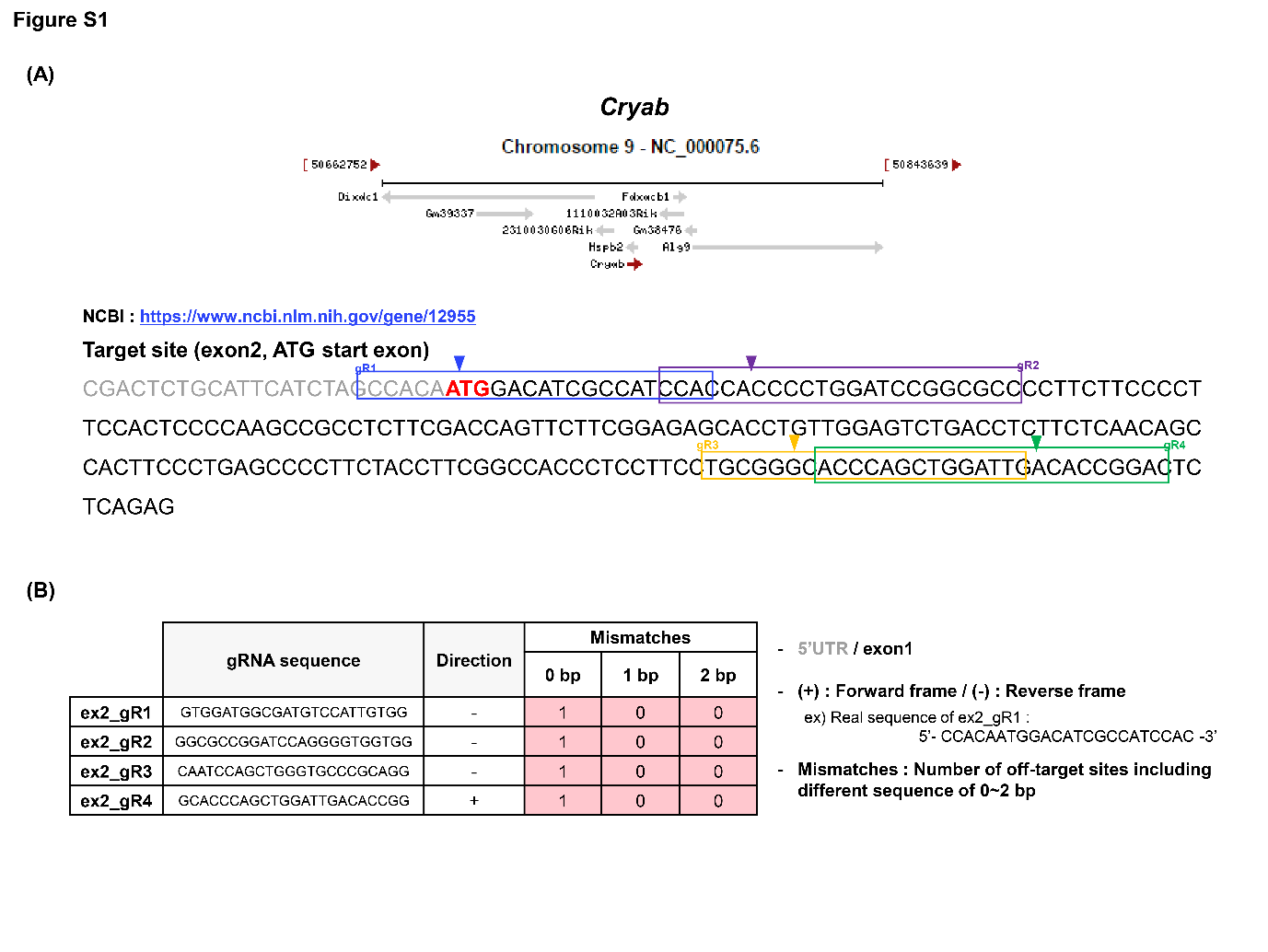


**Supporting information Figure S1** Altered Transcription site of exon in CRYAB KO mice. (A) Four types of gRNA in the ATG start exon target site. (B) Sequence of designed gRNAs. gRNA, guide RNA.


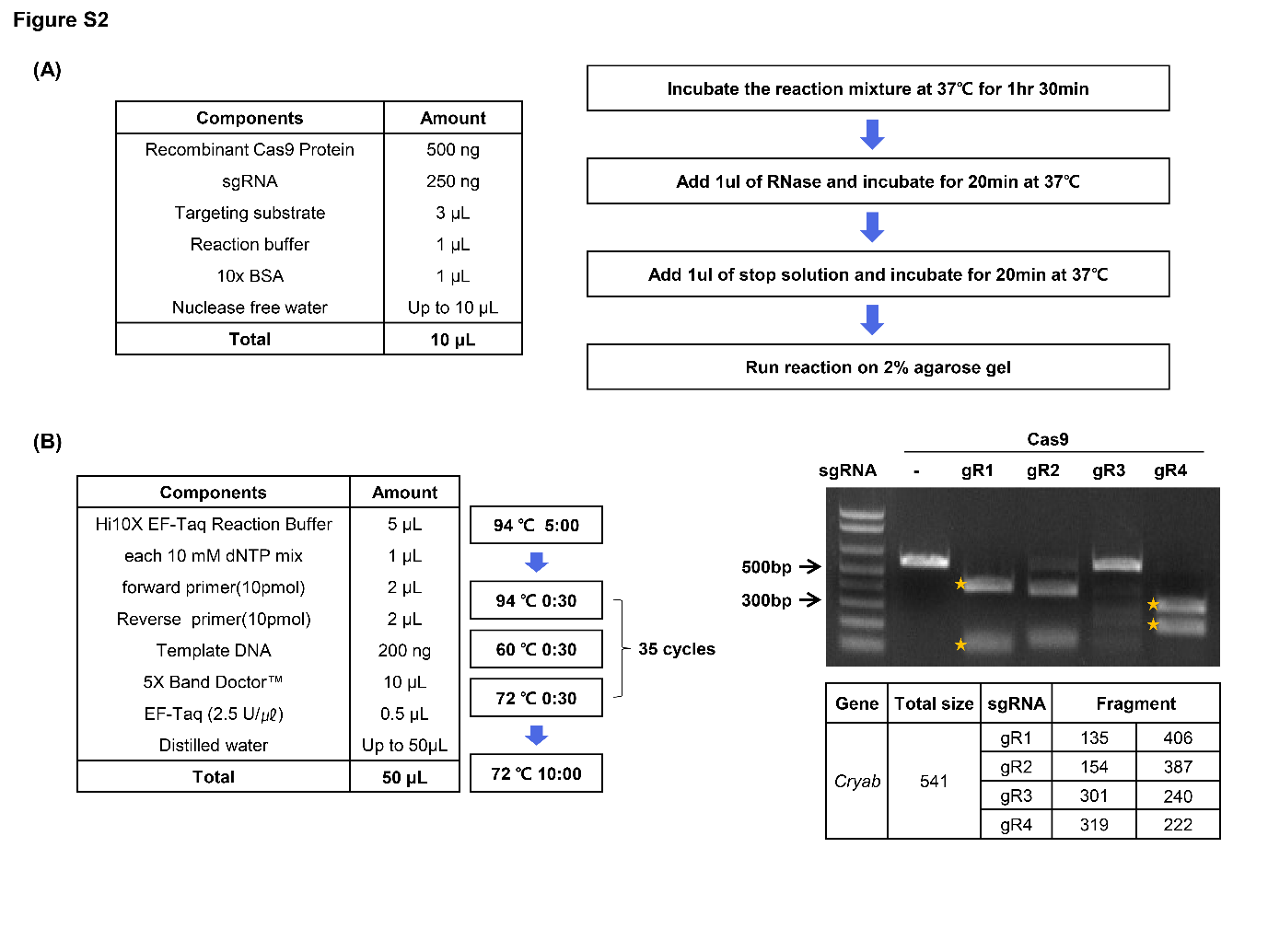


**Supporting information Figure S2** Materials and PCR conditions for CRISPR/Cas9 experiments. (A) Components and methods for the synthesis of sgRNA and Cas9 protein. (B) Components and PCR conditions for the confirmation of CRYAB cleaved form.


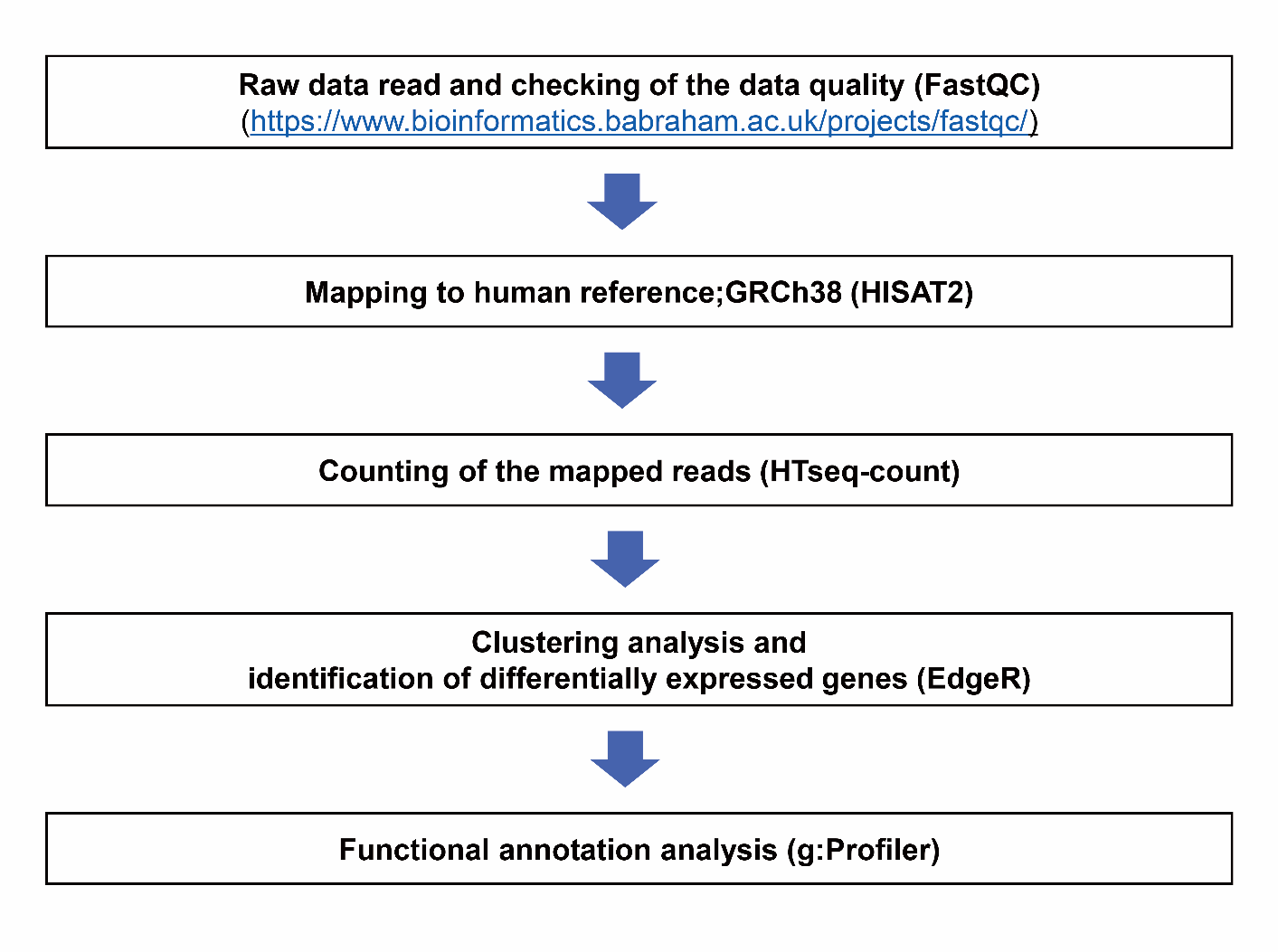


**Supporting information Figure S3** Human RNA-seq analysis process. Quality control of the raw FASTQ files, read mapping, and read quantification were conducted as previously described^1^ with the following modifications. To enhance accuracy and obtain comprehensive gene expression profiles, we used the GRCh38 reference genome assembly in HISAT2 to align our RNA-seq reads. Additionally, to identify and remove outlier samples from our RNA-seq dataset, we employed a clustering-based approach. This method resulted in the exclusion of one sample from the data analysis. The quality of raw data from all samples is initially checked using FastQC (https://www.bioinformatics.babraham.ac.uk/projects/fastqc/) and passed. Sequencing reads are mapped to the human reference genome, GRCh38, using HISAT2 (version 2.2.1)^3^. Counting of the mapped reads is performed using HTseq-count (subprogram of HTseq, version 2.0.1)^4^ with the GRCh38 annotation file, and no strand-specific and intersection-nonempty options. Next, we identified and adjusted for confounding variables in the normalized read count data using surrogate variable analysis (SVA package, version 3.50.0)^5^. Then, we conducted a clustering analysis on the adjusted read count data to detect outlier samples and the samples were excluded from the downstream analysis. The final differentially expressed genes (DEGs) analysis included RNA-Seq data from the NAcc of the 15 past users, 14 present users, and 49 controls. To identify the DEGs in the NAcc between drug users and controls, we compared the read counts of genes from both groups using a generalized linear model with surrogate covariates in EdgeR software (version 3.31.5)^6^. A false discovery rate (FDR) of < 0.05 is considered significant. For functional annotation of the DEGs, the Enrichment of Kyoto Encyclopedia of Genes and Genomes (KEGG) pathways enriched in these genes are identified using gProfiler^7^. The FDR is computed using the Benjamini–Hochberg method to correct the error rate of multiple testing. An FDR of < 0.05 is considered significant. DEG, differentially expressed gene; FDR, false discovery rate, HISAT2, hierarchical indexing for spliced alignment of transcripts; HT-seq, high-throughput sequencing; NAcc, nucleus accumbens.


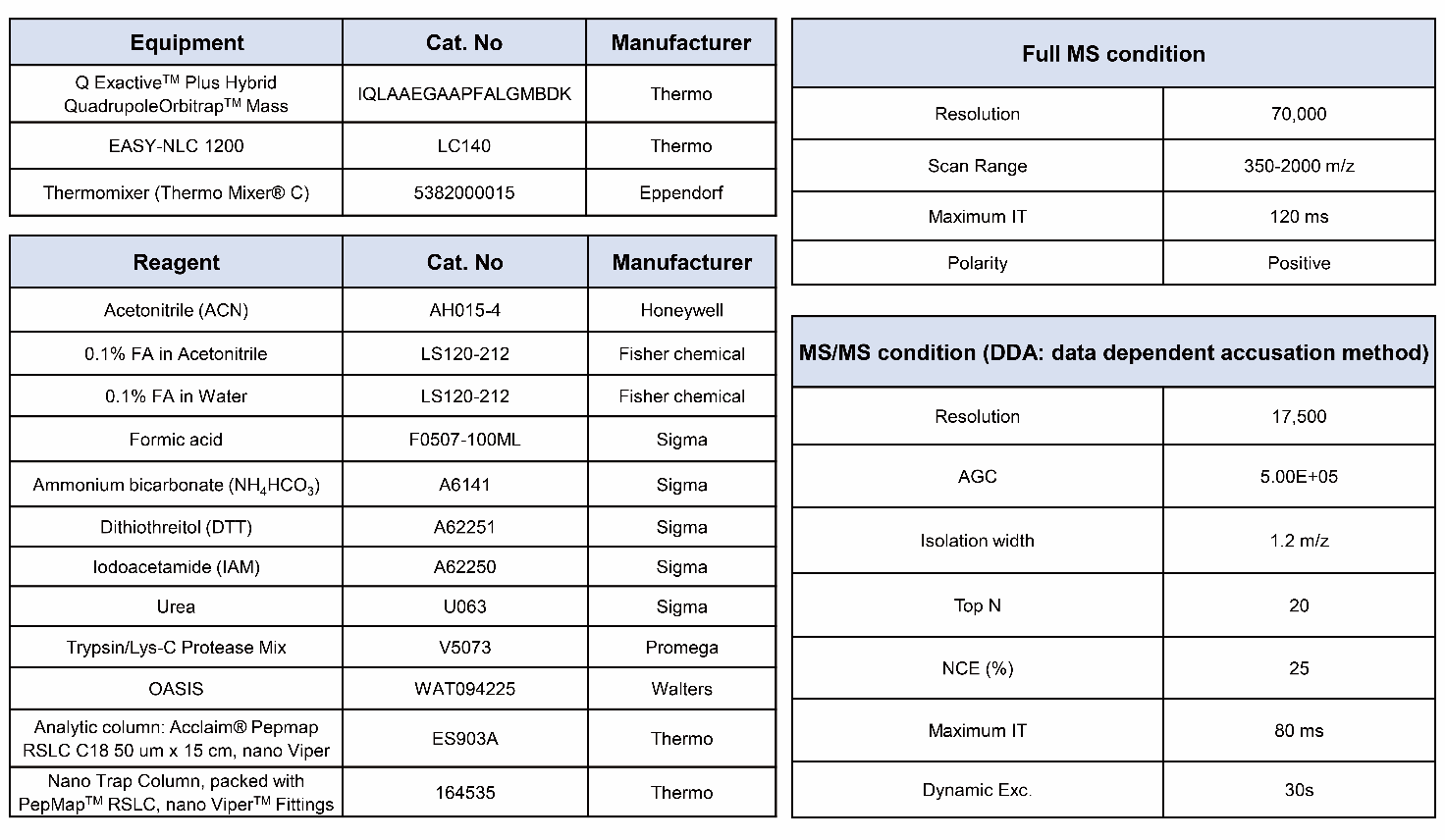


**Supporting information Figure S4** Equipment, reagents, and detailed conditions used for LC-MS/MS analysis.


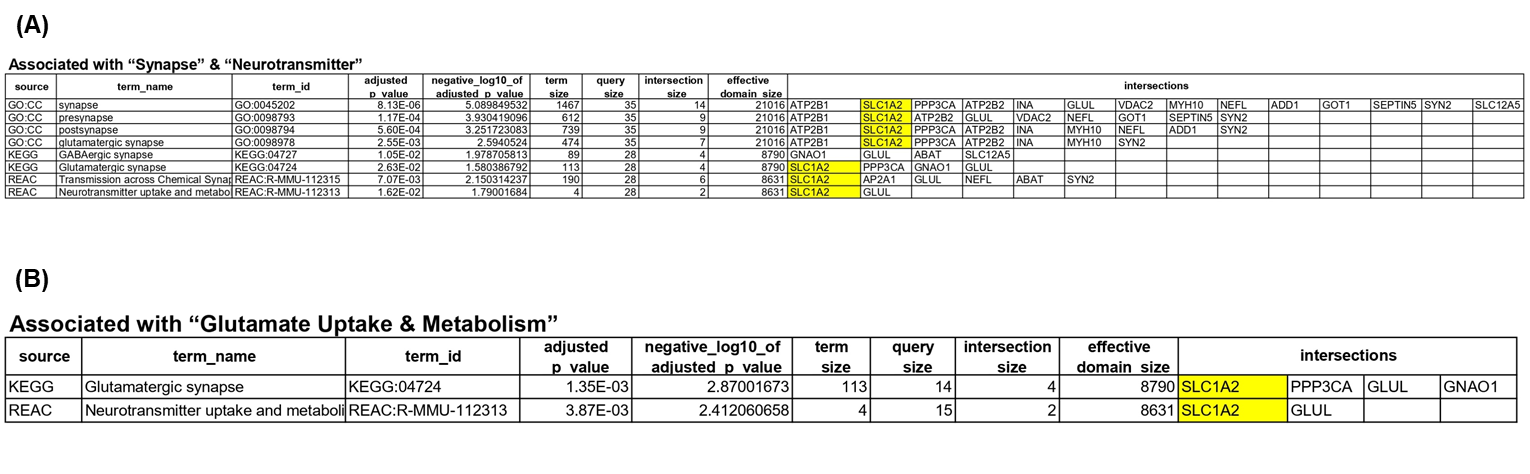


**Supporting information Figure S5** Omics analysis to find proteins associated with CRYAB. After analysis on increased proteins (logFC > 3.0) in cocaine group on LC-MS/MS data from CRYAB Co-IP sample (control and cocaine-treated group), analysis on GO, KEGG, and REAC using by g:Profiler (https://biit.cs.ut.ee/gprofiler/gost) were performed to find proteins associated with (A) synapse & neurotransmitter and (B) glutamate uptake & metabolism.


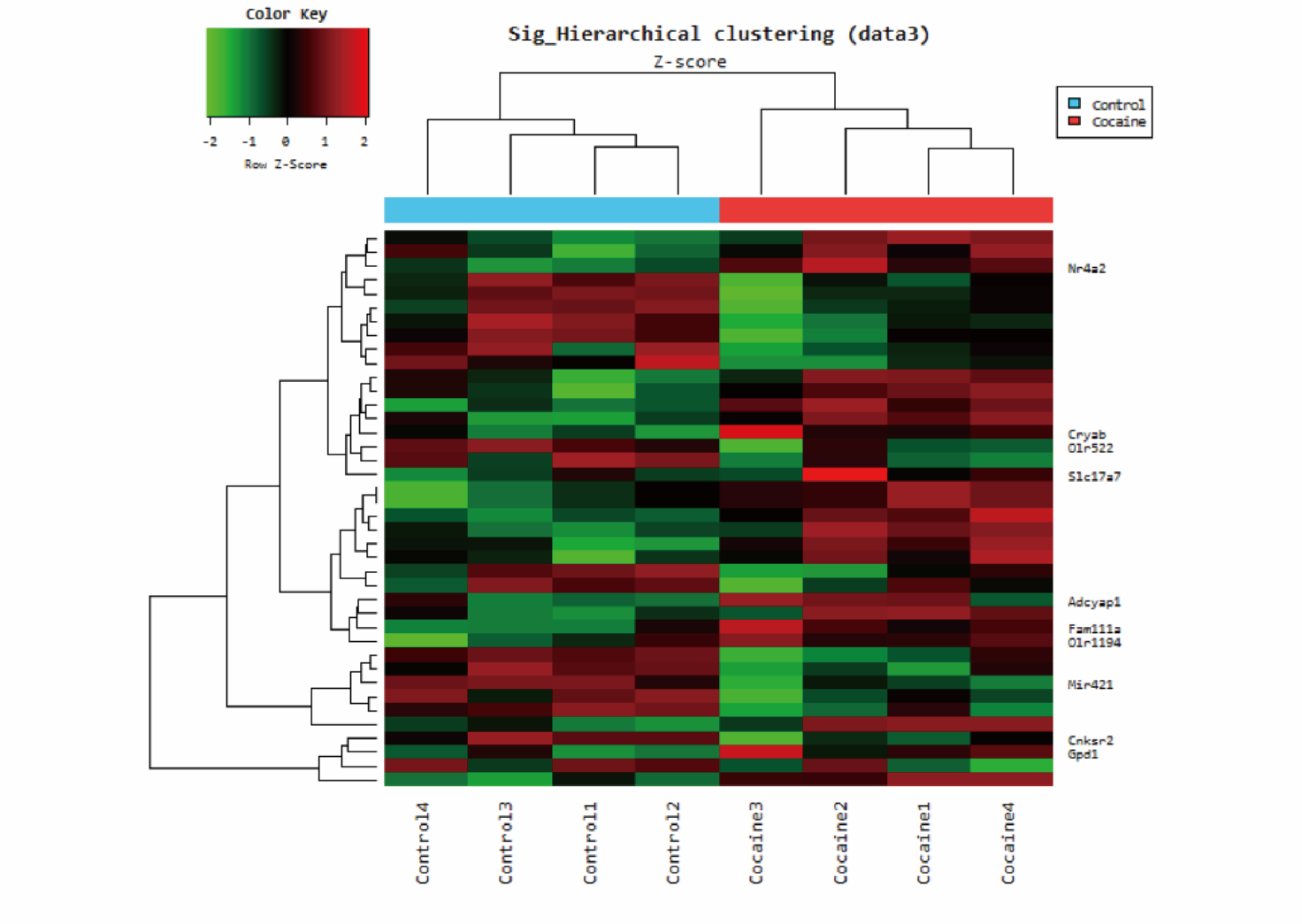


**Supporting information Figure S6** Hierarchical clustering of the detected genes from the DNA microarray assay. The cocaine-sensitized rats and the control group are sacrificed on the 8th day, after the locomotor test, and the NAcc was dissected for analysis. The microarray process is executed according to the manufacturer's protocol (GeneChipTH Whole Transcript PLUS reagent kit, 902280). We exported the results of gene-level RMA analysis and performed DEG analysis. The FDR is controlled by adjusting the p-value using the Benjamini–Hochberg algorithm. For a DEG set, hierarchical cluster analysis is performed using complete linkage and the Euclidean distance as a measure of similarity. Gene enrichment and functional annotation analysis for the significant probe list are performed using DAVID (http://david.abcc.ncifcrf.gov/home.jsp). All data analyses and visualization of the differentially expressed genes are conducted using R 3.1.2 (www.r-project.org). DEG, differentially expressed gene; FDR, false discovery rate; NAcc, nucleus accumbens; RMA, robust multi-array average.


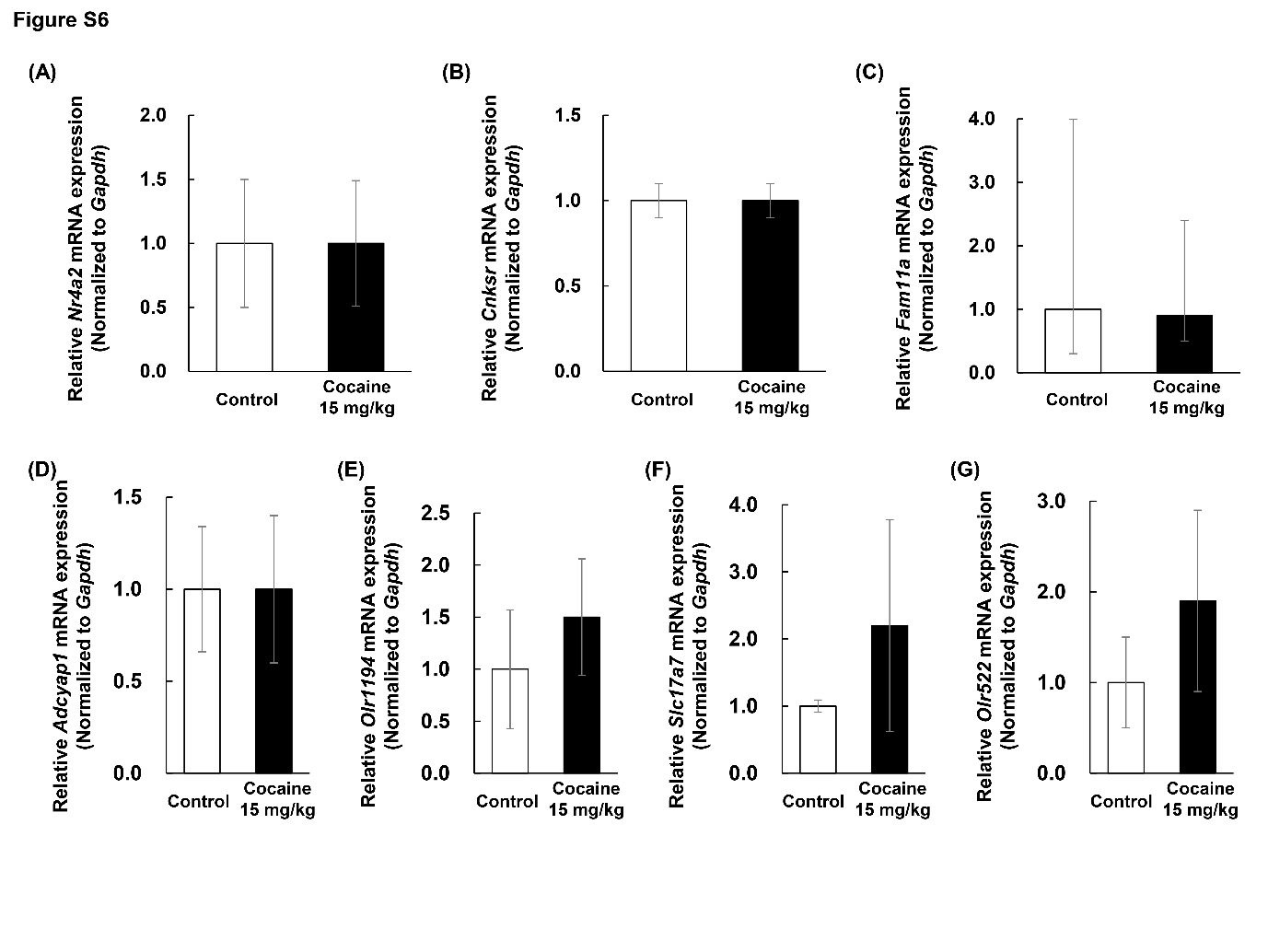


**Supporting information Figure S7** mRNA expression level of the target genes upon cocaine administration. (A-G) The mRNA expression level of the target genes detected in the microarray is confirmed using qPCR with each specific primer. The expression of each gene is normalized to the relative amplification of each *Gapdh*. Data are expressed as the mean ± S.E. (*n* = 4, Student’s t-test). The p-values for each gene are as follows: (A) 0.9365, (B) 0.9485, (C) 0.9695, (D) 0.8060, (E) 0.4385, (F) 0.3365, and (G) 0.3035.


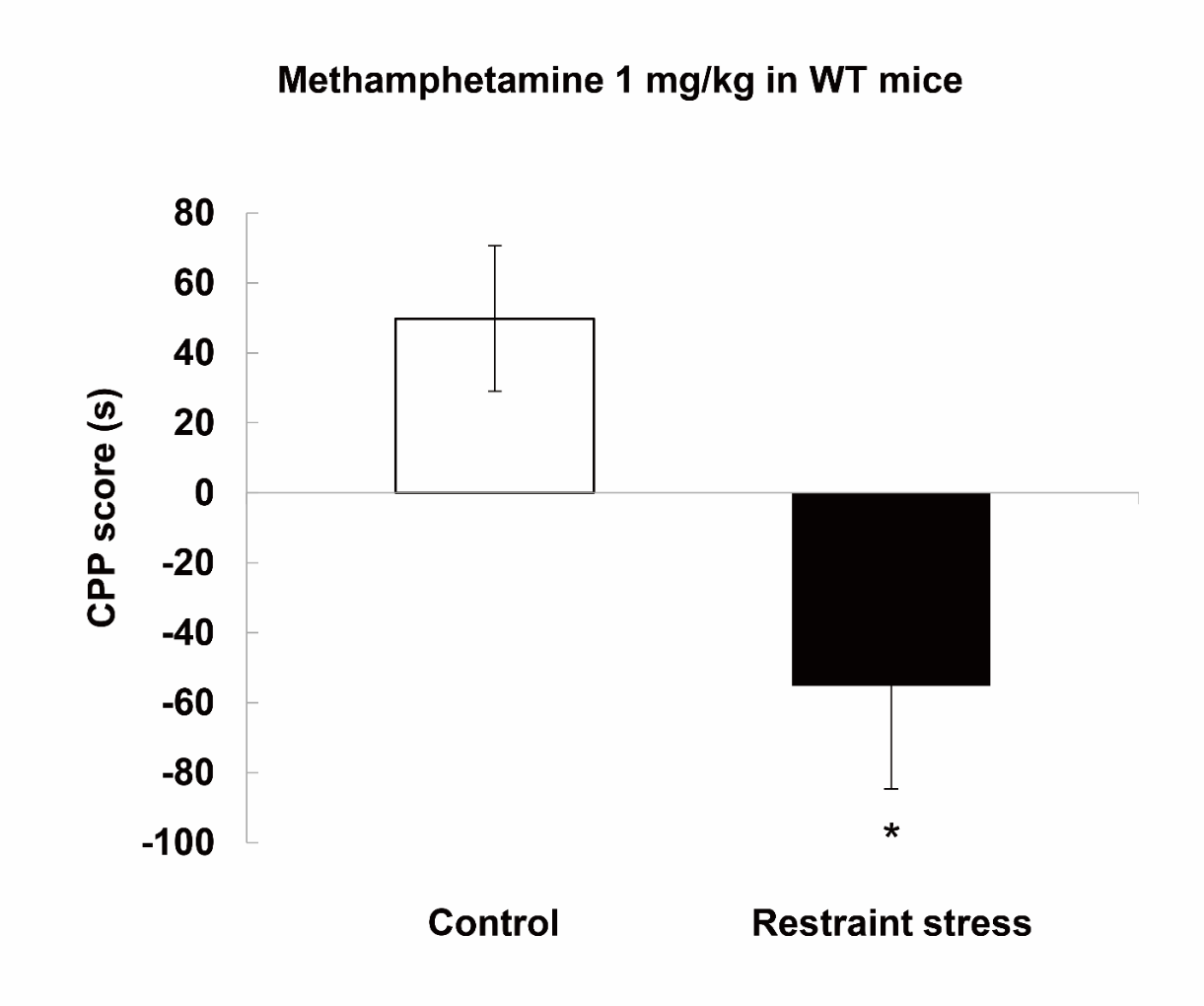


**Supporting information Figure S8** Effect of restrain stress on methamphetamine (1 mg/kg)-induced CPP development. Restraint stress is applied for 30 min by restraining the mouse in a 50-mL conical tube. The conditioning phase is then immediately commenced. Data are expressed as the mean ± S.E. (*n* = 4) and are analyzed using Student’s t-test (^*^*p* = 0.027 vs. control).


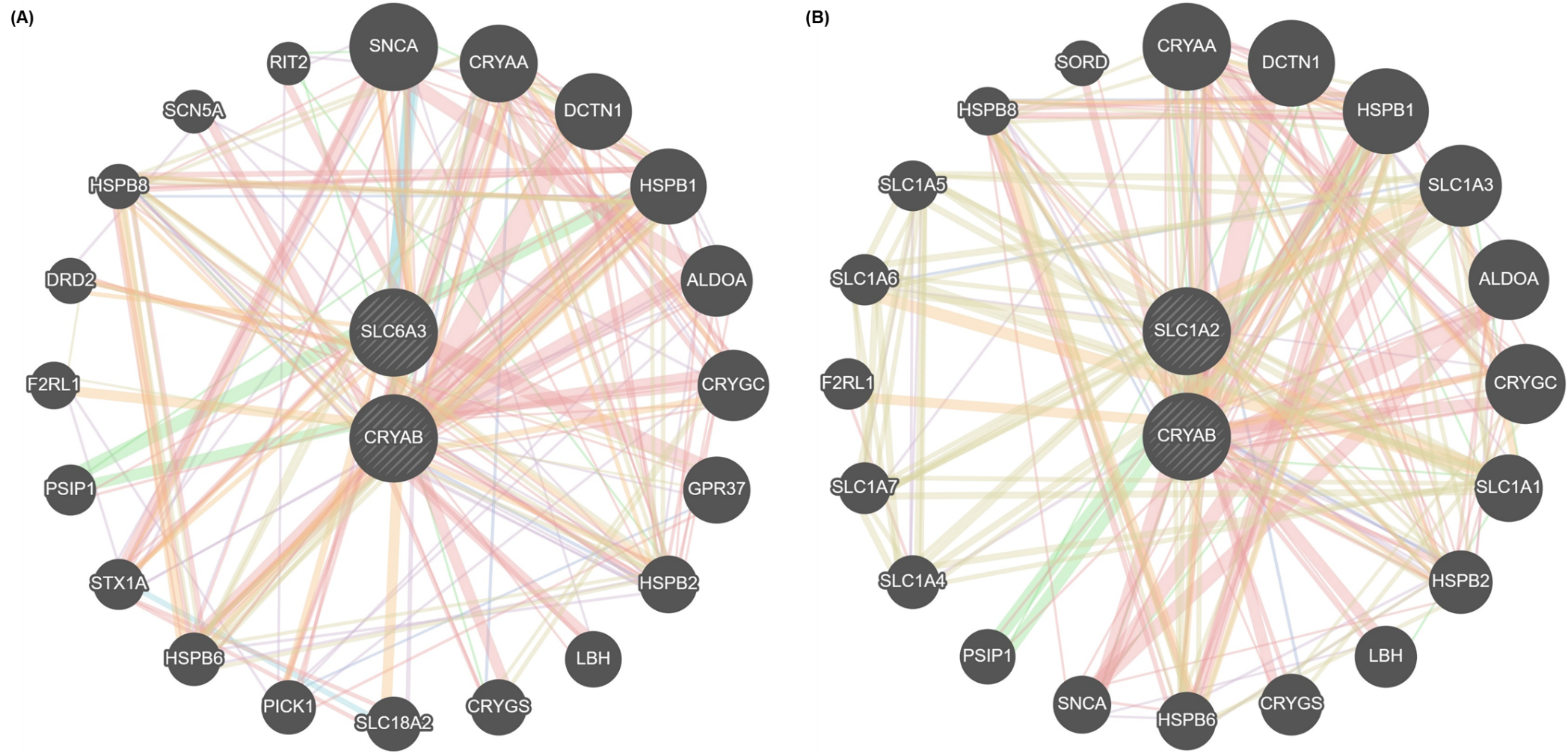


**Supporting information Figure S9** Analysis on gene–gene interaction with CRYAB. Interaction map showing the reference-based correlation between CRYAB (CRYAB coding gene) and (A) SLC1A2 [glutamate transporter 1 (EAAT2) coding gene] or (B) SLC6A3 [dopamine transporter (DAT) coding gene] using GeneMANIA (http://genemania.org/).


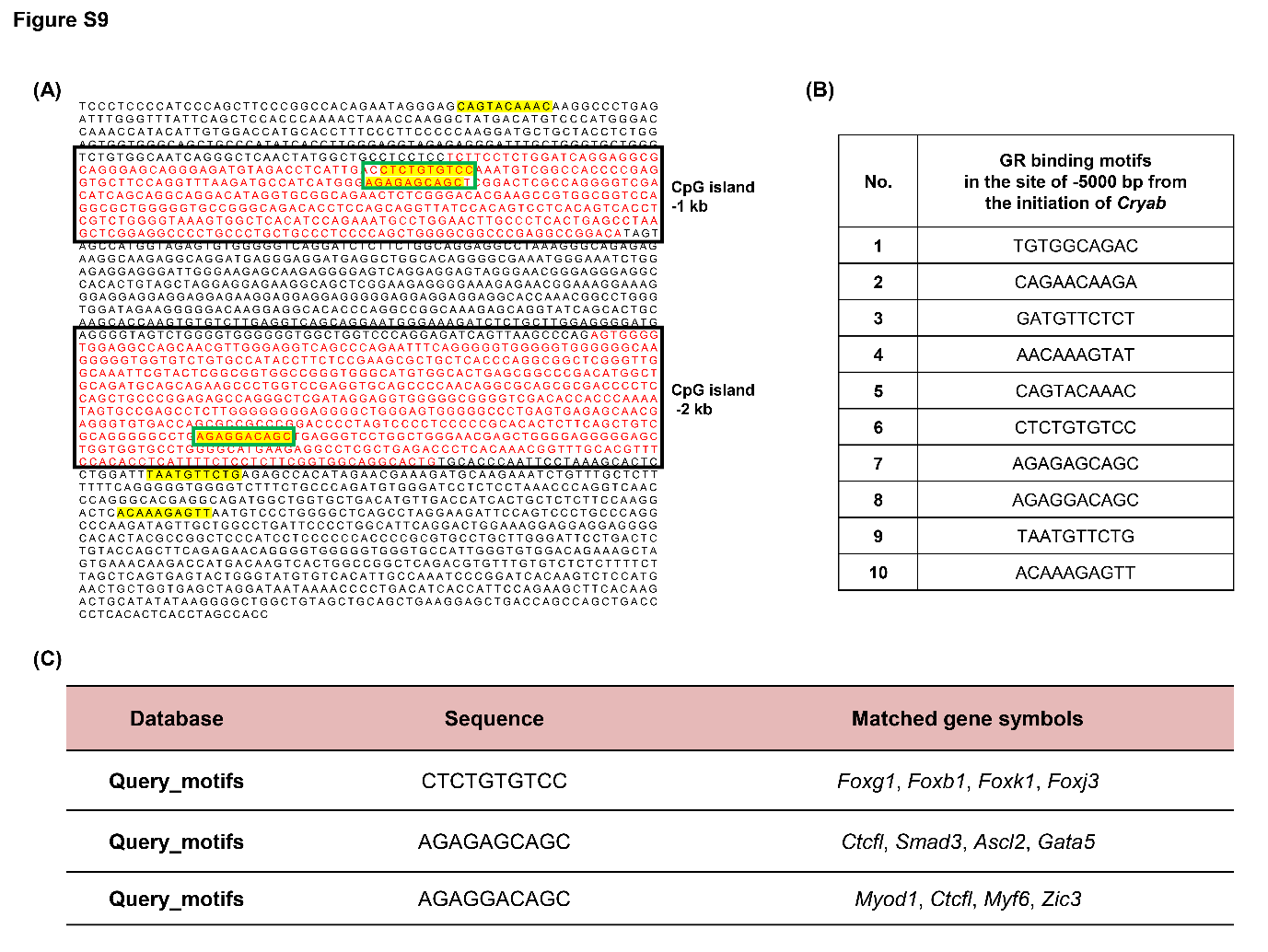


**Supporting information Figure S10** *Cryab* promotor region includes GRE binding sites. (A) The -2 kb sites of the *Cryab* promotor include a CpG island, where is a 5'-CG-3' rich region of DNA. The CpG island region includes GR motifs (red square). (B) GR-binding motifs in the site at –5,000 bp from the initiation of the *Cryab*. (C) GR motif-binding genes enhancing *Cryab* transcription in humans. CG, cytosine and guanine; CpG, 5'—C—phosphate—G—3'; GR, glucocorticoid receptor.


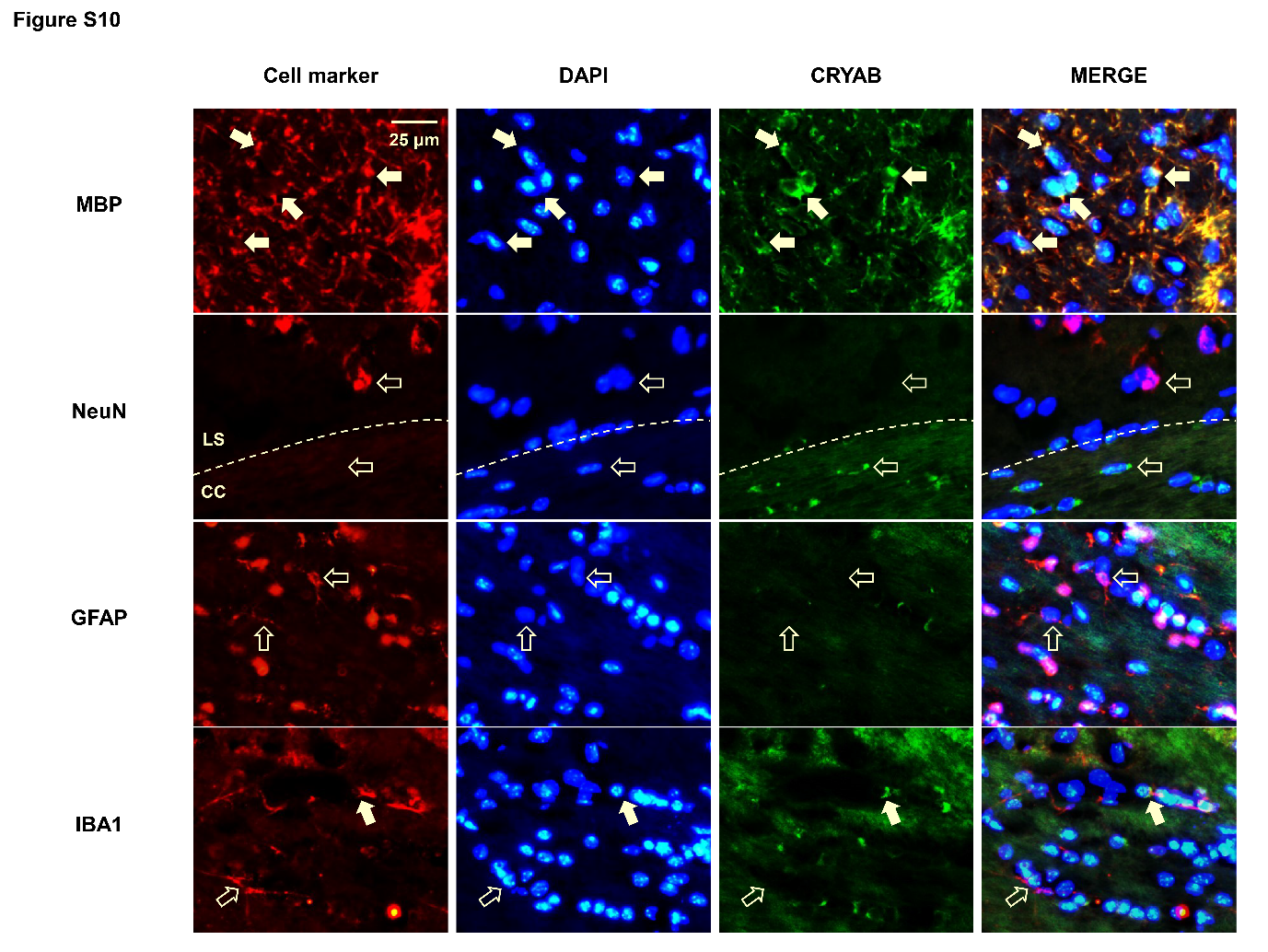


**Supporting information Figure S11** CRYAB-expressing cell types in the cocaine-administered mice corpus callosum. The mice are injected with cocaine 5 mg/kg (i.p.). The mice brain sections (10-μm-thick) are reacted with anti-CRYAB (green) and anti-MBP, anti-NeuN, anti-GFAP, and anti-IBA1 (brain cell markers; red). MBP, NeuN, GFAP, and IBA1 are markers of oligodendrocytes, neurons, astrocytes, and microglia, respectively. Closed arrows indicate the co-localization of each marker and CRYAB. Open arrows indicate the non-co-localization of each marker and CRYAB. The original magnification is ×200. The scale bar is 25 μm. CC, corpus callosum; LS, lateral septal nucleus.

**References**

1. Kim S, Jo Y, Webster MJ, Lee D. Shared co-expression networks in frontal cortex of the normal aged brain and schizophrenia. *Schizophr Res.* 2019;204:253-261.

2. Kim S, Webster MJ. The Stanley Neuropathology Consortium Integrative Database (SNCID) for Psychiatric Disorders. *Neurosci Bull.* 2019;35(2):277-282.

3. Kim D, Paggi JM, Park C, Bennett C, Salzberg SL. Graph-based genome alignment and genotyping with HISAT2 and HISAT-genotype. *Nat Biotechnol.* 2019;37(8):907-915.

4. Anders S, Pyl PT, Huber W. HTSeq--a Python framework to work with high-throughput sequencing data. *Bioinformatics.* 2015;31(2):166-169.

5. Leek JT, Storey JD. Capturing heterogeneity in gene expression studies by surrogate variable analysis. *PLoS Genet.* 2007;3(9):1724-1735.

6. Robinson MD, McCarthy DJ, Smyth GK. edgeR: a Bioconductor package for differential expression analysis of digital gene expression data. *Bioinformatics.* 2010;26(1):139-140.

7. Raudvere U, Kolberg L, Kuzmin I, et al. g:Profiler: a web server for functional enrichment analysis and conversions of gene lists (2019 update). *Nucleic Acids Res.* 2019;47(W1):W191-W198.
